# Effect of different methods of cooling for targeted temperature management on outcome after cardiac arrest: a systematic review and meta-analysis

**DOI:** 10.1186/s13054-019-2567-6

**Published:** 2019-08-23

**Authors:** Lorenzo Calabró, Wulfran Bougouin, Alain Cariou, Chiara De Fazio, Markus Skrifvars, Eldar Soreide, Jacques Creteur, Hans Kirkegaard, Stéphane Legriel, Jean-Baptiste Lascarrou, Bruno Megarbane, Nicolas Deye, Fabio Silvio Taccone

**Affiliations:** 1 Department of Intensive Care, Cliniques Universitaires de Bruxelles Hopital Erasme, Erasmus Hospital, Université Libre de Bruxelles (ULB), Route de Lennik, 808, 1070 Brussels, Belgium; 2 UFR de Médecine, Université Paris-Descartes-Sorbonne-Paris-Cité, Paris, France; 3 Paris Sudden Death Expertise Center, Paris, France; 4 Paris Cardiovascular Research Center, INSERM U970, Paris, France; 5 Medical ICU, Cochin Hospital, Assistance Publique-Hôpitaux de Paris, Paris, France; 6 Division of Intensive Care, Department of Anesthesiology, Intensive Care and Pain Medicine, Helsinki University Hospital and Helsinki University, Helsinki, Finland; 7 Department of Anaesthesiology and Intensive Care, Stavanger University Hospital, Stavanger, Norway; 8 Research Center for Emergency Medicine, Department of Anesthesiology and Intensive Care Medicine, Aarhus University Hospital and Aarhus University, Aarhus, Denmark; 9 Intensive Care Unit, Centre Hospitalier de Versailles, PARCC Inserm UMR 970, Le Chesnay, France; 10 Medical Intensive Care Unit, University Hospital Center, PARCC Inserm UMR 970, Nantes, France; 11 Medical and Toxicology Intensive Care Unit, Hôpitaux Universitaires Saint Louis-Lariboisière, Assistance Publique-Hôpitaux de Paris, Université Paris Diderot-Paris 7, Inserm U942, Paris, France

**Keywords:** Targeted temperature management, Methods, Endovascular, Surface cooling, Survival, Neurological outcome, Meta-analysis

## Abstract

**Background:**

Although targeted temperature management (TTM) is recommended in comatose survivors after cardiac arrest (CA), the optimal method to deliver TTM remains unknown. We performed a meta-analysis to evaluate the effects of different TTM methods on survival and neurological outcome after adult CA.

**Methods:**

We searched on the MEDLINE/PubMed database until 22 February 2019 for comparative studies that evaluated at least two different TTM methods in CA patients. Data were extracted independently by two authors. We used the Newcastle-Ottawa Scale and a modified Cochrane ROB tools for assessing the risk of bias of each study. The primary outcome was the occurrence of unfavorable neurological outcome (UO); secondary outcomes included overall mortality.

**Results:**

Our search identified 6886 studies; 22 studies (*n* = 8027 patients) were included in the final analysis. When compared to surface cooling, core methods showed a lower probability of UO (OR 0.85 [95% CIs 0.75–0.96]; *p* = 0.008) but not mortality (OR 0.88 [95% CIs 0.62–1.25]; *p* = 0.21). No significant heterogeneity was observed among studies. However, these effects were observed in the analyses of non-RCTs. A significant lower probability of both UO and mortality were observed when invasive TTM methods were compared to non-invasive TTM methods and when temperature feedback devices (TFD) were compared to non-TFD methods. These results were significant particularly in non-RCTs.

**Conclusions:**

Although existing literature is mostly based on retrospective or prospective studies, specific TTM methods (i.e., core, invasive, and with TFD) were associated with a lower probability of poor neurological outcome when compared to other methods in adult CA survivors (CRD42019111021).

**Electronic supplementary material:**

The online version of this article (10.1186/s13054-019-2567-6) contains supplementary material, which is available to authorized users.

## Introduction

Effective neuroprotective strategies are required to prevent or minimize the development of extended anoxic brain injury after cardiac arrest (CA) after the return of spontaneous circulation (ROSC) [1]. The use of targeted temperature management (TTM) is actually recommended in comatose CA survivors, especially after out-of-hospital cardiac arrest (OHCA) with an initial shockable rhythm, although the quality of evidence is moderate to low [2]. In 2002, two randomized clinical trials (RCTs) showed that TTM at 33 °C for 12–24 h was associated with a significantly higher proportion of patients achieving a favorable neurological outcome when compared to any temperature control [3–5]; more recently, a large RCT showed that the target temperature during TTM could be either 33 °C or 36 °C, with an active temperature control required for all patients [6].

Current guidelines recognized some important knowledge gaps in the use of TTM after CA. In particular, although significant delay to initiate TTM could negate the positive effects of such intervention, RCTs showed no benefits from early TTM initiation using cold intravenous fluids, either during cardiopulmonary resuscitation (CPR) or immediately after ROSC, when compared to in-hospital TTM implementation [7, 8]. Also, the optimal TTM duration remains unknown; while prolonged therapy up to 72 h is effective in newborns suffering from anoxic-hypoxic encephalopathy [9], TTM at 33 °C for 48 h did not significantly improve long-term neurological outcome when compared to 24 h duration in adult OHCA [10]. Importantly, all these studies dealing with early or prolonged cooling strategies suffered from significant biases and might have been underpowered. As such, these aspects of TTM after CA, together with some other issues, such as the selection of patients (i.e., shockable vs. non-shockable patients, in-hospital vs. OHCA), the rewarming rate, and the control of post-TTM fever, have not been adequately addressed yet.

Moreover, the most effective method to deliver TTM and how this could influence the patients’ outcome is a matter of debate. According to international guidelines, external or internal cooling devices can be used [2]. Several devices are available for clinicians, with different technical characteristics, possibilities of use (i.e., in-hospital vs. out-of-hospital), velocity to achieve target temperature, precision (i.e., maintenance of the target temperature within target ranges), invasiveness, potential side effects, and costs [11]. Furthermore, basic means (such as ice packs, cold fluid, fans) seem of less precision and efficiency than automated method (i.e., automatic adjustment according to patient temperature) [12]. To date, no RCTs showed the superiority of a specific device over another [12, 13]. However, most studies found that endovascular cooling devices (EC) enabled a more precise temperature control when compared to others [14, 15] and some of them also reported a non-significant trend towards a better survival rate [16, 17].

The aim of this metanalysis was therefore to investigate whether, in patients resuscitated from cardiac arrest (i.e., participants) undergoing TTM (i.e., intervention), neurological outcome and survival (i.e., Outcomes) could be influenced by the different methods used for TTM (i.e., comparison).

## Methods

We adhered to the *Preferred Reporting Items for Systematic Reviews and Meta-Analysis-Protocols* (PRISMA-P) guidelines [18]. The protocol of this study was registered with the *International Prospective Register of Systematic Reviews* (PROSPERO) on 25 January 2019 and finally approved on 12 February 2019 (CRD42019111021).

### Data sources and search strategies

A systematic literature search was performed up to 30 January 2019 in the MEDLINE/PubMed® database. This approach may have reduced the number of eligible citations; however, other reference libraries have a higher proportion of non-English citations, with probably smaller cohorts of patients for the TTM setting. However, as one study was already accepted for publication at that moment [19], the literature search was extended up to 22 February 2019.

This search included only original studies published in English in peer-reviewed journals. The search was performed using the following terms: (“hypothermia” OR “TTM” OR “cooling” OR “cooling method” OR “targeted temperature management”) AND (“heart arrest” OR “cardiac arrest” OR “post-anoxic”). In addition, we also searched the reference lists of all eligible studies as well as relevant reviews for additional published and unpublished data, searched by contacting experts, and used a web search for abstracts, proceedings, and unpublished studies. More details regarding data collection are presented in Additional file 1: Table S1. Main research questions, with reference to participants, interventions, comparisons, outcomes, and study design (PICOS), are reported in Additional file 1: Table S1.

### Study screening and selection

The studies were independently screened by two authors (LC and CDF), looking at the study titles and abstracts for potential eligibility. Additional citations were also identified by the authors of the present review based on their prior knowledge of the literature. Disagreement between the authors was assessed and resolved through a third reviewer (WB), who reviewed the original text of the article. In the analysis, we included only the studies that compared at least two cooling methods (i.e., one vs. the other) in adult (> 18 years of age) CA patients; studies could be either retrospective, prospective, or RCTs. Studies conducted in healthy volunteers or in animal/experimental models were excluded. Editorials, commentaries, letters to the editor, opinion articles, reviews, meeting abstracts, case reports, and studies published in other languages were excluded; all original articles lacking an abstract and/or quantitative details on neurological outcome and survival were also excluded. None of the authors of the original studies was contacted to obtain further information, as the main outcomes were all clearly stated in the published manuscript.

From this first pool of selected articles, further criteria were established for the inclusion in the quantitative analysis: (a) TTM should be used in both groups (i.e., both methods should be used to induce hypothermia at least at 36 °C or below); (b) when more than one cooling method was simultaneously used for a group, the group was classified according to the most frequently used cooling method used (i.e., if in one group, 90% of patients receive “method A” and 25% “method B,” the groups will be considered as being treated with “method A”); (c) if one of the methods was used only for a limited time period (i.e., only for the induction of TTM) and then discontinued, the study was excluded; and (d) if TTM was attempted using only antipyretics or intravenous drugs (i.e., such as neurotensin, clonidine), the study was also excluded.

### Definitions

Cooling methods were classified as “core” (i.e., EC, intravenous cold fluids, automated peritoneal lavage, any dialysis technique, extra-corporeal membrane oxygenation, esophageal or trans-nasal) or “surface” (i.e., skin exposure, cooling beds, iced packs, cooling pads, air-circulating or water-circulating blankets, water-filled blankets, air-filled blankets) [11]. Additional classifications included “invasive” (i.e., EC, automated peritoneal lavage, any dialysis technique, extra-corporeal membrane oxygenation) or “non-invasive” (all the others) and “temperature feedback devices” (TFD, i.e., with a controlled feedback system that continuously measures the patients’ temperature and adjust the temperature of the cooling element accordingly) or “non-TFD.” If a study included multiple methods, the data were aggregated prior to inclusion in the systematic review according to the abovementioned definitions (i.e., EC and esophageal cooling devices would be considered together as “core,” but EC will eventually be included in the “invasive” group while esophageal cooling devices in the “non-invasive” group).

### Appraisal of study quality

The level of evidence (LOE) of each study was assessed according to the *Grading of Recommendations, Assessment, Development and Evaluations* (GRADE) evidence system [20]. The risk of bias for RCTs was assessed using a modified Cochrane ROB tool that classifies ROB as “low,” “probably low,” “probably high,” or “high” for each of the following domains: sequence generation, allocation sequence concealment, blinding, selective outcome reporting, and other bias [21]. The risk of bias of non-RCTs was assessed using the Newcastle-Ottawa Scale [22]; in particular, we evaluated three components: (a) selection of cases: studies were considered as “low” ROB if case definition was adequate, cases were representative, and outcome of interest was not present at the beginning of the study; (b) comparability of cohorts: studies were considered as “low” ROB if adjustment was made for usual prognostic factors (i.e., Utstein variables); (c) exposure and outcome: studies were considered as “low” ROB if assessment of outcome and follow-up were appropriate. Overall, a study was considered as “low” ROB if each single component was classified as “low.” LOE was further analyzed by two experts (WB, ND) and one independent statistician. Disagreement was resolved by consensus.

### Primary and secondary outcomes

The primary outcome evaluated was the analysis of unfavorable neurological outcome, whenever this was collected and defined as cerebral performance category of 3–5, in the “core” vs. “surface” group. The secondary outcome was mortality, whenever this was collected. Similar subgroup analyses were performed in invasive vs. non-invasive methods and TFD vs. non-TFD methods. Pre-defined subgroup analyses were performed in (a) EC vs. surface, (b) EC vs. surface with TFD, (c) surface blankets vs. other surface methods, as EC and blanket cooling devices, are the most used methods in this setting worldwide.

### Statistical analysis

Means of mortality and poor neurological outcome risks were obtained by weighting each study by the inverse of variance. Mantel-Haenszel method was chosen as the reference method for fixed effects analysis. The Mantel-Haenszel formula is applied to calculate an overall, unconfounded, effect estimate of a given exposure for a specific outcome by combining stratum-specific odds ratios (OR). Stratum-specific ORs are calculated within each stratum of the confounding variable and compared with the corresponding effect estimates in the whole group. A *Z* test was carried out to assess the significance of the risk differences. The *I*^2^ was calculated by *χ*^2^ test to assess the variability due to heterogeneity rather than chance. A substantial heterogeneity was assumed with *I*^2^ > 50%. 95% CI for mortality and neurologic outcome were calculated with the Wilson method and placed in forest plots, and statistical significance was assumed for *p* < 0.05. The presence of publication bias was evaluated by trim and fill. The trim and fill method estimates the number of missing studies from a meta-analysis due to the suppression of the most extreme results on one side of the funnel plot. Then, this method augments the observed data and recomputes the summary estimate based on the complete data. The trim and fill outputs were obtained with iterations. Analyses were performed for all the selected studies, as well as grouped by RCT vs. observational trials. Statistical analysis was conducted by Review Manager 5.3 software, and funnel and forest plots were developed.

## Results

### Study selection

**Fig. 1:**
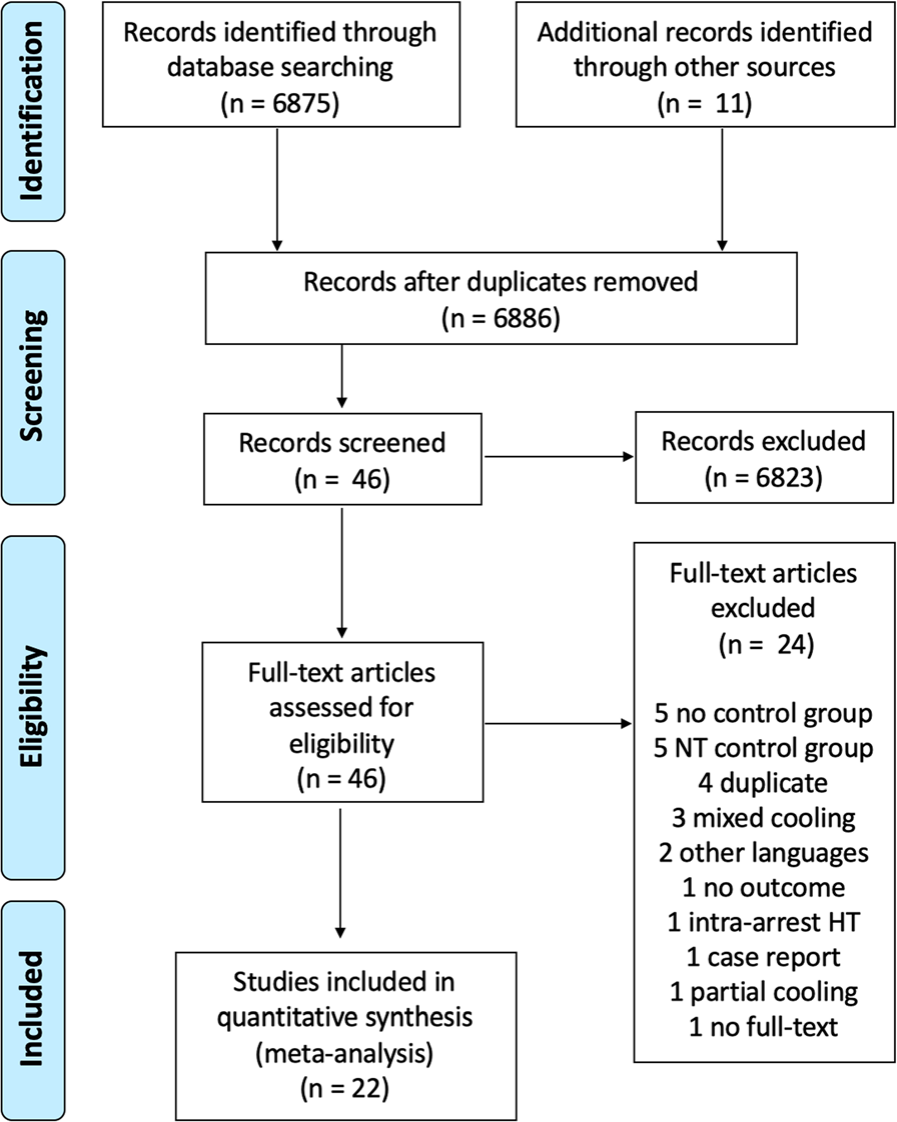
PRISMA flow diagram of the search results for original studies published in English and selection of eligible studies. NT, normothermia; HT, hypothermia

A total of 6886 records were identified after the initial research. After the first screening procedure, 46 studies were assessed for eligibility (Fig. 1). Of those, 24 were excluded after a full-paper analysis (Additional file 1: Table S2); a total of 22 studies [12–19, 23–37], including 8027 patients, were eventually included for meta-analysis.

### Study characteristics

The characteristics of the selected studies are summarized in Table 1 and Additional file 1. We identified 4 RCTs (high quality of evidence for two [12, 33] and moderate for two [13, 24]; Additional file 1: Table S3), four prospective studies (low level of evidence [16, 27, 35, 37] with an additional one comparing a prospective cohort with historical controls (very low quality of evidence [36]), and 13 retrospective studies (very low quality of evidence [15, 17, 23, 25, 26, 28–32, 34]), with two of them being a secondary post hoc analysis of RCTs (low quality of evidence [14, 19]). The risk of bias (RB) was low in one study [14] and high for all the others (Tables 2 and 3).

**Table 1:** Summary of studies comparing different methods for targeted temperature management (TTM) in adult cardiac arrest patients

Author, year [ref]	Study	OHCA/IHCA	Patients	Target temperature	Duration TTM	Mortality	Neurological outcome	TTM method 1 (*n*), TFD (Y/N)	TTM method 2 (*n*), TFD (Y/N)	LOE
Look, 2018 [13]	RCT	OHCA	45	32–34 °C	24 h	Hospital discharge	Hospital discharge	Endovascular (23), Y	Blankets (22), Y	Moderate
Glover, 2016 [14]	R*	OHCA	934	33 or 36 °C	24 h	6 months	6 months	Endovascular (240), Y	Surface (694), Y	Low
Deye, 2015 [12]	RCT	OHCA	400	32–34 °C	24 h	3 months	28 days	Endovascular (203), Y	Surface (197), N	High
Oh, 2015 [15]	R	OHCA	803	32–34 °C	24 h	Hospital discharge	Hospital discharge	Endovascular (244), Y	Surface (559), Y	Very low
De Waard, 2015 [23]	R	OHCA	173	32–34 °C	24 h	Hospital discharge	NR	Endovascular (97), Y	Blankets (76), Y	Very low
Pittl, 2013 [24]	RCT	Both	80	32–34 °C	24 h	Hospital discharge	Hospital discharge	Endovascular (39), Y	Blankets (39), Y	Moderate
Tomte, 2011 [17]	R	OHCA	167	32–34 °C	24 h	6 months	6 months	Endovascular (75), Y	Blankets (92), Y	Very low
Flint, 2007 [31]	R	Both	42	32–34 °C	24 h	Hospital discharge	NR	Endovascular (19), Y	Blankets (23), Y	Very low
Sonder, 2018 [16]	P	Both	120	32–34 °C	24 h	Hospital discharge	Hospital discharge	Endovascular (48), Y	Blankets (72), Y	Low
Gillies, 2010 [32]	R	Both	83	32–34 °C	12 to 24 h	Hospital discharge	Hospital discharge	Endovascular (42), Y	Blankets (41), N	Very low
Ferreira, 2009 [28]	R	OHCA	49	32–34 °C	24 h	Hospital discharge	Hospital discharge	Endovascular (24), Y	Surface (25), N	Very low
Flemming, 2006 [30]	R	OHCA	80	32–34 °C	24 h	Hospital discharge	NR	Endovascular (31), Y	Blankets (49), Y	Very low
Rosman, 2016 [25]	R	Both	34	32–34 °C	24 h	ICU discharge	NR	Endovascular (17), Y	Blankets (17), N	Very low
Caulfield, 2011 [27]	P	Both	41	32–34 °C	24 h	Undefined	NR	Endovascular (26), Y	Blankets (15), Y	Low
De Fazio, 2019 [19]	R*	OHCA	352	32–34 °C	24 or 48 h	6 months	6 months	Endovascular (218), Y	Blankets (134), Y	Low
Feuchtl, 2007 [29]	R	OHCA	39	32–34 °C	24 h	Hospital discharge	NR	Endovascular (19), Y	Cold packs (20), N	Very low
Shinada, 2014 [34]	R	Both	51	32–34 °C	24 h	1 month	1 month	Blanket (40), Y	Blankets (11), Y	Very low
Rana, 2011 [35]	P	OHCA	46	32–34 °C	24 h	Hospital discharge	NR	Blanket (28), N	Cold fluids + packs (18), N	Low
Heard, 2010 [33]	RCT	OHCA	64	32–34 °C	24 h	Undefined	3 months	Blanket (34), Y	Blankets (30), N	High
De Waard, 2013 [36]	P**	Both	115	32–34 °C	24 h	ICU discharge	NR	Peritoneal lavage (16), Y	Blankets (99), Y	Very low
Kim, 2018 [26]	R	Both	4246	32–34 °C	24 h	Hospital discharge	Hospital discharge	Endovascular (376), Y Intra-cavitary (377), N	Blankets (2107), Y Blankets, pads, packs (1386), N	Very low
Forkmann, 2015 [37]	P	OHCA	63	32–34 °C	24 h	30-day	NR	Endovascular (40), Y	Blankets (23), N	Low

*P* prospective, *R* retrospective, *RCT* randomized clinical trial, *IHCA* in-hospital cardiac arrest, *OHCA* out-of-hospital cardiac arrest, *TTM* targeted temperature management, *NR* not reported, *LOE* level of evidence, *TFD* temperature feedback device, *Y* yes, *N* no

*Post hoc analysis of RCT

**Versus historical controls

**Table 2:** Summary of the risk of bias (ROB) for non-randomized clinical studies comparing different methods for targeted temperature management (TTM) in adult cardiac arrest patients. LOW ROB = 0; HIGH ROB = 1

Author, year [ref]	Selection of cases	Comparability of cohorts	Exposure and outcome	Overall ROB
Glover, 2016 [14]	1	1	1	High
Oh, 2015 [15]	1	1	0	High
De Waard, 2015 [23]	1	0	0	High
Tomte, 2011 [17]	1	0	1	High
Flint, 2007 [31]	1	0	0	High
Sonder, 2018 [16]	1	0	0	High
Gillies, 2010 [32]	1	0	0	High
Ferreira, 2009 [28]	1	0	0	High
Flemming, 2006 [30]	1	0	0	High
Rosman, 2016 [25]	1	1	0	High
Caulfield, 2011 [27]	1	0	0	High
De Fazio, 2019 [19]	1	0	0	High
Feuchtl, 2007 [29]	1	1	0	High
Shinada, 2014 [34]	1	1	1	High
Rana, 2011 [35]	1	1	0	High
De Waard, 2013 [36]	1	0	0	High
Kim, 2018 [26]	1	0	1	High
Forkmann, 2015 [37]	1	1	1	High

**Table 3:** Summary of the risk of bias (ROB) for randomized clinical studies comparing different methods for targeted temperature management (TTM) in adult cardiac arrest patients

Author, year [ref]	Sequence generation	Allocation concealment	Blinding	Incomplete data	Selective reporting	Other bias	Overall ROB
Look, 2018 [13]	Low	Low	Probably low	Probably low	Low	Probably high	High
Deye, 2015 [12]	Low	Low	Probably low	Low	Low	Low	Low
Pittl, 2013 [24]	Probably low	Probably low	Probably low	Probably low	High	High	High
Heard, 2010 [33]	Low	Low	Probably low	Probably low	Low	Probably high	High

### Core vs. surface

**Fig. 2:**
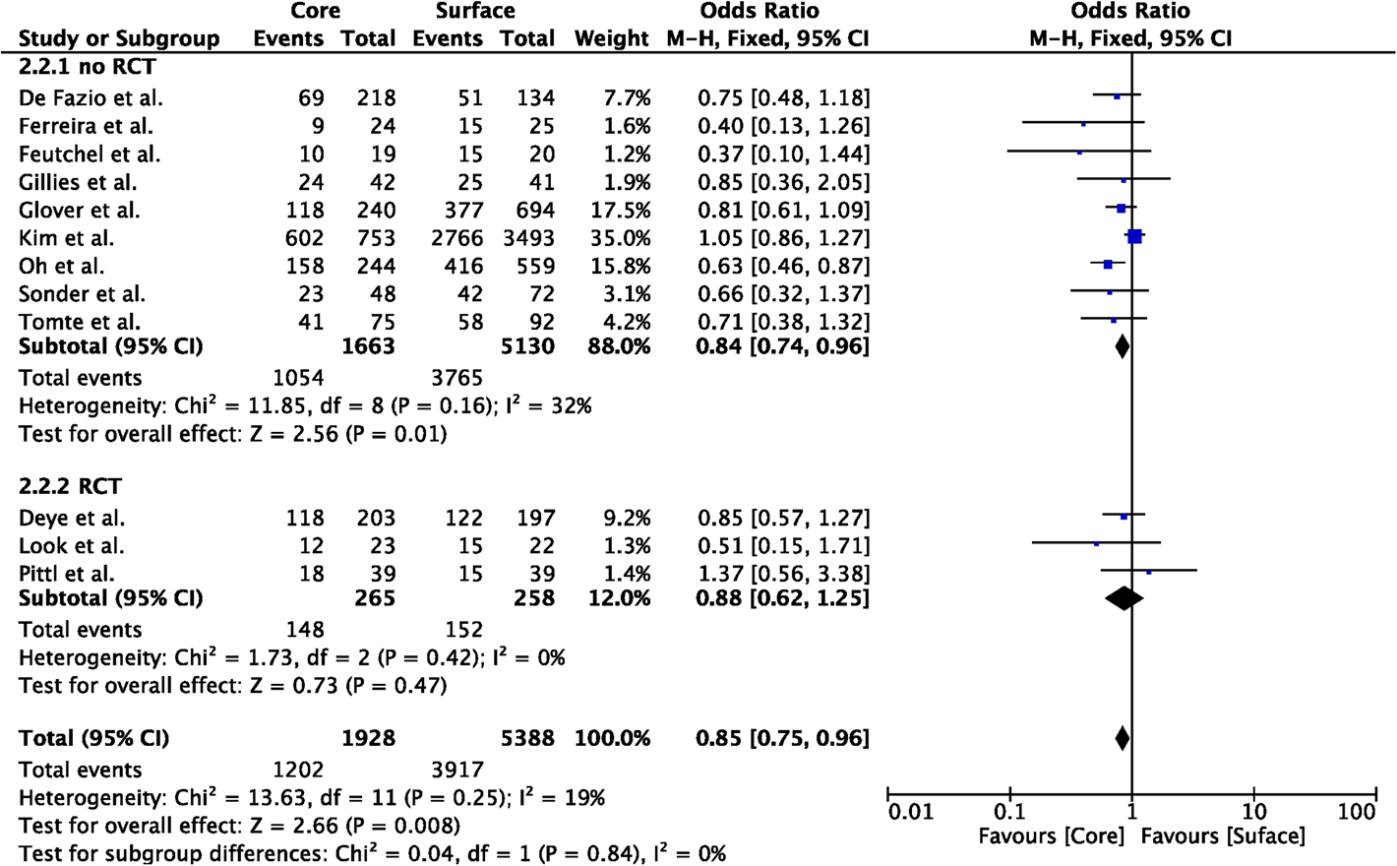
Forest plot of poor neurological outcome in randomized clinical trials (RCTs) or non-RCTs: core vs. surface methods. The size of the squares for the risk ratio reflects the weight of trial in a pooled analysis. Horizontal bars represent 95% confidence intervals

**Fig. 3:**
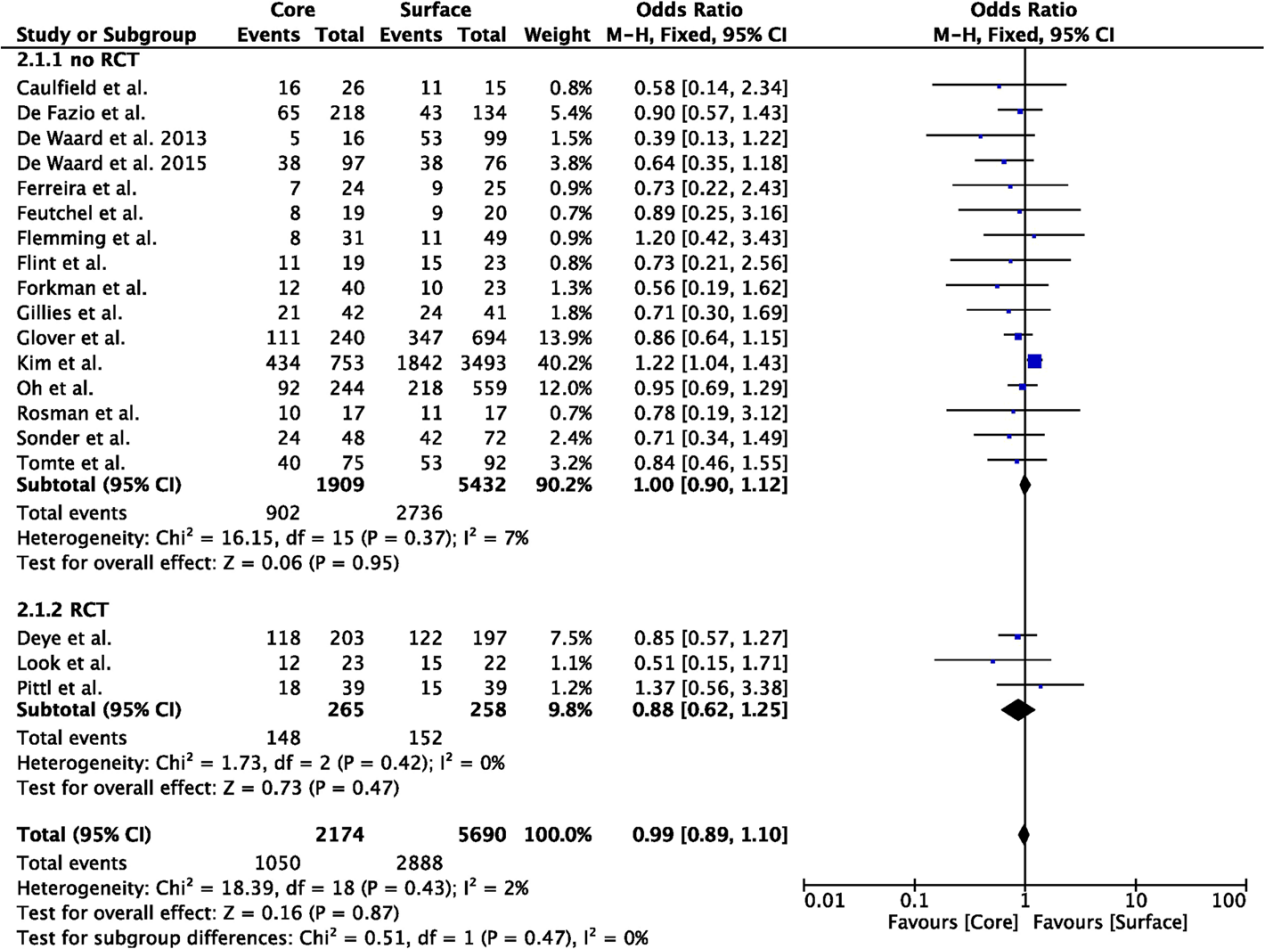
Forest plot of poor neurological outcome in randomized clinical trials (RCTs) or non-RCTs: core vs. surface methods. The size of the squares for the risk ratio reflects the weight of trial in a pooled analysis. Horizontal bars represent 95% confidence intervals

Nineteen studies compared core to surface TTM methods (*n* = 2174 in the core group; *n* = 5690 in the surface group) [12–17, 19, 23–32, 36, 37]; unfavorable neurological outcome was reported in 12 studies (*n* = 1928 in the core group; *n* = 5388 in the surface group) [12–17, 19, 25, 26, 28, 29, 32]. Core methods showed a lower probability of unfavorable neurological outcome than surface methods (OR 0.85 [95% CIs 0.75–0.96]; *p* = 0.008; Fig. 2); however, this was observed in the analysis of non-RCTs, although RCTs showed a similar trend. Core methods showed a similar probability of mortality than surface methods (OR 0.88 [95% CIs 0.62–1.25]; *p* = 0.21; Fig. 3). No significant heterogeneity was observed among studies, both for unfavorable neurological outcome (*I*^2^ = 19%) and mortality (*I*^2^ = 0%; Additional file 1: Figures S1 and S2).

### Invasive vs. non-invasive

**Fig. 4:**
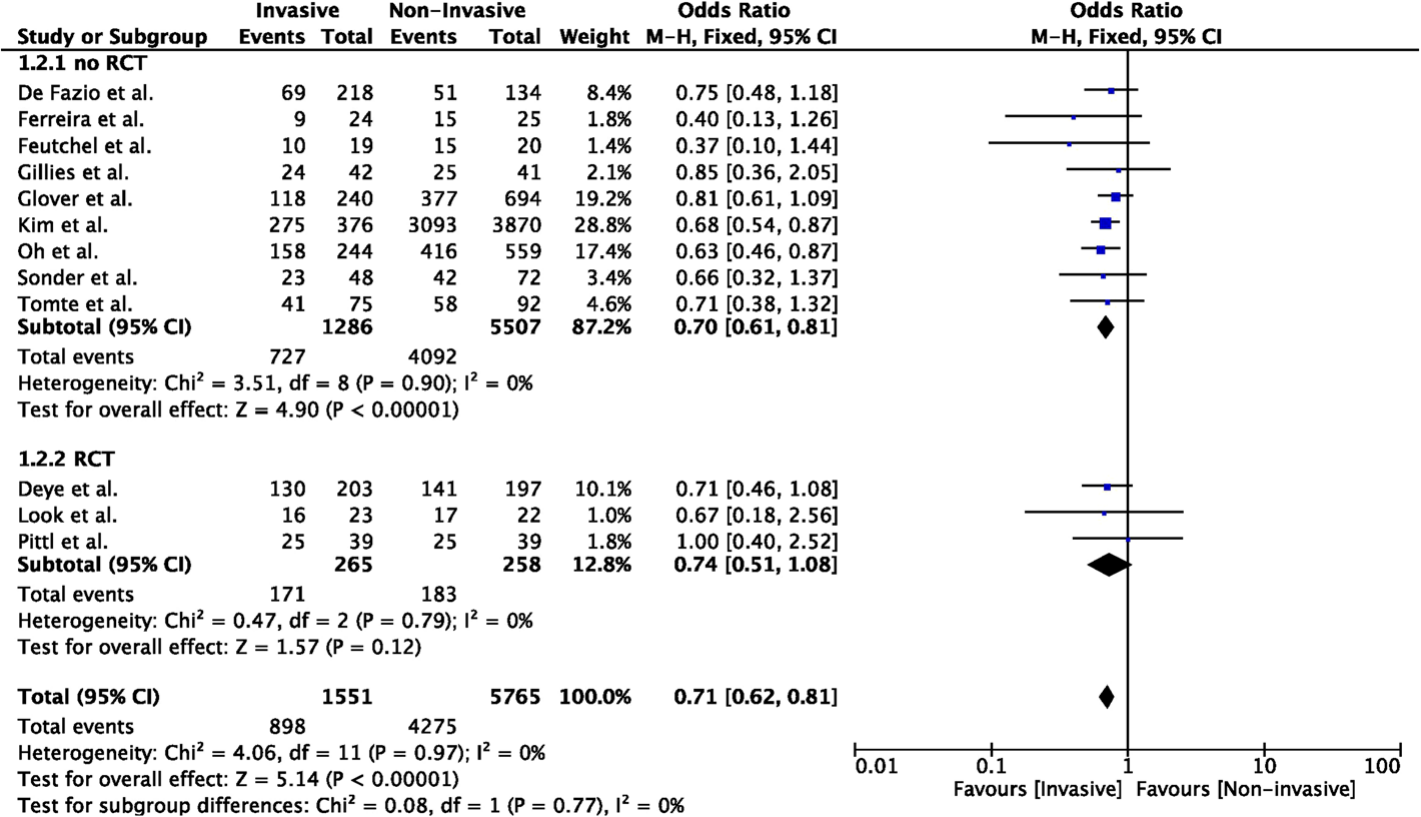
Forest plot of poor neurological outcome in randomized clinical trials (RCTs) or non-RCTs: invasive vs. non-invasive methods. The size of the squares for the risk ratio reflects the weight of trial in a pooled analysis. Horizontal bars represent 95% confidence intervals

Nineteen studies compared invasive to non-invasive TTM methods (*n* = 1797 in the invasive group; *n* = 6067 in the non-invasive group) [12–17, 19, 23–32, 36, 37]; unfavorable neurological outcome was reported in 12 studies (*n* = 1551 in the invasive group; *n* = 5765 in the non-invasive group) [12–17, 19, 24, 26, 28, 29, 32]. Invasive methods showed a lower probability of unfavorable neurological outcome than non-invasive methods (OR 0.70 [95% CIs 0.61–0.81]; *p* < 0.001; Fig. 4); however, this was observed in the analysis of non-RCTs, although RCTs showed a similar trend. Invasive methods showed also a lower probability of mortality than non-invasive methods (OR 0.84 [95% CIs 0.74–0.94]; *p* = 0.002; Additional file 1: Figure S3); however, this was observed only in the analysis of non-RCTs (*n* = 13). No significant heterogeneity was observed among studies, both for unfavorable neurological outcome (*I*^2^ = 0%) and mortality (*I*^2^ = 0%; Additional file 1: Figures S4 and S5).

### TFD vs. no-TFD

**Fig. 5:**
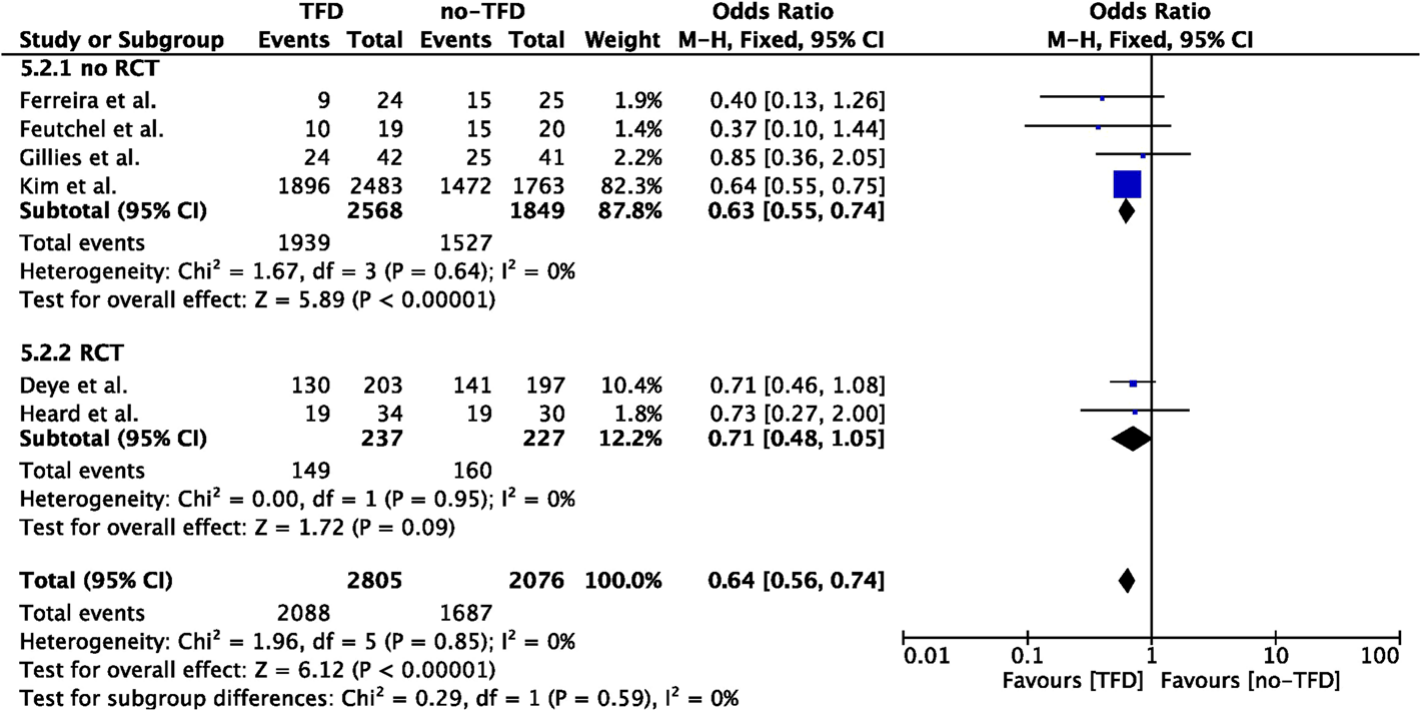
Forest plot of poor neurological outcome in randomized clinical trials (RCTs) or non-RCTs: temperature feedback devices (TFD) vs. non-TFD. The size of the squares for the risk ratio reflects the weight of trial in a pooled analysis. Horizontal bars represent 95% confidence intervals

Eight studies compared TFD to non-TFD TTM methods (*n* = 2862 in the TFD group; *n* = 2116 in the non-TFD group) [12, 25, 26, 28, 29, 32, 33, 37]; unfavorable neurological outcome was reported in 6 studies (*n* = 2865 in the TFD group; *n* = 2076 in the non-TFD group) [12, 26, 28, 29, 32, 33]. TFD methods showed a lower probability of unfavorable neurological outcome than non-TFD methods (OR 0.64 [95% CIs 0.56–0.74]; *p* = 0.003; Fig. 5); however, this was observed in the analysis of non-RCTs, although RCTs showed a similar trend. Invasive methods showed also a lower probability of mortality than surface methods (OR 0.81 [95% CIs 0.72–0.91]; *p* = 0.01; Additional file 1: Figure S6). No significant heterogeneity was observed among the studies, both for unfavorable neurological outcome (*I*^2^ = 0%) and mortality (*I*^2^ = 0%; Additional file 1: Figures S7 and S8).

### Subgroup analyses

Sixteen studies compared EC to air- or water-circulating blankets for TTM [13–17, 19, 23–28, 30–32, 37]; ten of them also reported neurological outcome [13–17, 19, 24, 26, 28, 32]. Endovascular methods showed a lower probability of unfavorable neurological outcome than air- or water-circulating blankets (OR 0.76 [95% CIs 0.66–0.87]; *p* = 0.002; Additional file 1: Figure S9); however, this was observed in the analysis of non-RCTs, although RCTs showed a similar trend. Endovascular methods showed also a lower probability of mortality than air- or water-circulating blankets (OR 0.87 [95% CIs 0.77–0.99]; *p* = 0.01; Additional file 1: Figure S10); however, this was observed in the analysis of non-RCTs, although RCTs showed a similar trend. No significant heterogeneity was observed among studies, both for unfavorable neurological outcome (*I*^2^ = 0%) and mortality (*I*^2^ = 0%; Additional file 1: Figures S11 and S12). Similar results were found when EC were compared to air- or water-circulating blankets with TFD (Additional file 1: Figures S13-S16) [13–17, 19, 23, 24, 26, 27, 30, 31]. Four studies compared air- or water-circulating blankets to other surface TTM methods [26, 33–35]; three of them also reported neurological outcome [26, 33, 34]. Air- or water-circulating blankets showed a lower probability of unfavorable neurological outcome and mortality than other surface TTM methods (Additional file 1: Figures S17 and S18). No significant heterogeneity was observed among studies, both for unfavorable neurological outcome (*I*^2^ = 0%) and mortality (*I*^2^ = 0%; Additional file 1: Figure S19 and S20).

## Discussion

This is the first meta-analysis that evaluated the potential effects of the cooling methods on the outcome in patients undergoing TTM after cardiac arrest. Our results can be summarized as follows: core cooling devices are associated with a lower probability of unfavorable neurological outcome, but not of survival, when compared to surface cooling devices; similarly, invasive cooling devices and TFD methods were associated with a higher occurrence of favorable outcome and survival when compared to other methods. In particular, endovascular catheter devices were associated with better outcomes when compared to air- or water-circulating blankets, even when blankets were TFD, and blankets had a better outcome than other surface cooling methods. However, most of the available data came from non-RCTs, potentially leading to high risk of bias.

### Method of cooling: which is the best TTM device?

The initial studies dealing with the use of TTM after cardiac arrest used external surface cooling devices, either with TFD or without temperature control. Since then, several TTM methods to induce, maintain, or rewarm patients have been developed; these methods have been schematically divided into “core” or “surface” methods [11], although other classifications have been proposed, i.e., “advanced” methods (using a retro-control according to the patient’s core temperature) vs. “basic” methods (such as cold fluids, ice packs without temperature feedback) [12, 17, 38]. Core and TFD methods enable a more accurate control of patients’ temperature during the cooling phase when compared with surface techniques [12, 17, 19]; nevertheless, current guidelines stated that no recommendation could be given on the optimal method, as there were no data indicating any potential benefits on survival or neurological outcome from one specific TTM device [2]. The use of TFD to better optimize TTM was also recently suggested by a panel of French experts [39], in both adult and pediatric CA, despite the lack of studies clearly demonstrating an effect on the patients’ outcome. Moreover, more recent invasive TTM devices, such as cold liquid ventilation and esophageal or peritoneal devices, were not included in this consensus.

A major strength of our study is that we demonstrated that core, invasive, and/or TFD methods to provide TTM to CA survivors were associated with an increased probability of favorable neurological outcome. These findings were consistent even in the subgroup analyses, when EC were compared to blanket systems or air- or water-circulating blankets to other surface methods. Moreover, the analyses showed a very low heterogeneity. Unfortunately, findings from RCTs were less convincing. In the largest multicenter RCT available to date, which compared EC to basic external methods (i.e., non-invasive, without TFD, *n* = 400), favorable neurological outcome at day 28 was not significantly different between the groups [12], although a strong trend towards a higher occurrence of intact neurological recovery at day 90 was observed in the EC group (odds ratio 1.51; 95% CIs 0.96–2.35; *p* = 0.07). In another study (*n* = 64), TTM using a TFD implementing water-circulating blankets showed a similar proportion of patients with favorable neurological outcome than cooling blankets and ice without TFD (46% vs. 38%); however, considering the total number of patients included, the study was probably underpowered to demonstrate any difference on the patients’ outcome [33]. Two other small RCTs, including a total of 125 patients, also showed a non-significant trend for a better outcome in patients treated with EC when compared to TFD blankets [13, 24].

How to explain these results? The limited data provided by the selected studies restricted our ability to explore the underlying mechanisms of our findings. Time to target temperature was significantly shorter in core and invasive TTM methods than others [12, 33], although this was not observed in all studies [14, 19]. Moreover, the early initiation of TTM in patients suffering from OHCA, either intra-arrest or immediately after ROSC in the pre-hospital setting, did not show any beneficial effects on survival or neurological outcome when compared to TTM initiated few hours following CA, after hospital admission [8, 40]. Analysis of the rewarming time showed controversial results, with some studies suggesting a shorter time for EC when compared to non-TFD blankets and other showing similar findings for EC and TFD surface methods [19, 25]. Also, the occurrence of post-TTM fever was similar between EC and surface methods [17, 19]. As such, the main difference between the core and invasive methods, in particular EC, was associated with a more strict maintenance of the target temperature during the cooling phase, fewer periods of over-cooling or unexpected rewarming and less temperature variability [14, 19, 25]. Importantly, temperature variability after CA has not been associated with poor neurological outcome in two retrospective studies [41, 42]. Considering also the higher risk of side effects (i.e., infections, thrombosis, hemorrhage) associated with the use of core and invasive TTM systems, in particular EC [12, 24, 43], further studies evaluating the mechanisms involved in potential neuroprotection for such methods are necessary.

### Major weaknesses

The results of this meta-analysis should be considered as a step forward a future trial evaluating the effects of cooling methods on the patients’ outcome, in order to better answer the question on the optimal method to provide TTM after CA. However, it remains unclear which methods should be compared in this study. Deye et al. [12] compared EC (i.e., core, invasive, TFD) with cooling blankets and ice packs (i.e., surface, non-invasive, and without TFD) and showed a non-significant but clinically relevant difference between the two groups. These findings are reinforced by this systematic review that observed a non-significant but large difference in the outcome when the two available RCTs comparing devices with TFD vs. those without TFD were considered. Whether it would be ethical to expose CA patients to TTM methods that are less effective in inducing hypothermia and maintaining a target temperature in such a trial, this should be further considered. The most adequate trial should compare EC with water-circulating blankets using TFD, as in the study from Pittl et al. [24]. Nevertheless, to reproduce the same differences in the neurological outcome (i.e., 53% vs. 61%), more than 1200 patients would be necessary. In the meantime, considering the cost and invasiveness of some of these devices, the selection of the best TTM strategy remains challenging; if one could argue that core and invasive TFD methods should be implemented in those patients with the best prognosis (i.e., young patient with a shockable initial rhythm), another could also suggest these methods on patients with the most severe reperfusion injuries (i.e., prolonged resuscitation with poor clinical presentation) in order to have more chance to prevent them. Also, it could also be considered unethical to use other methods than those with TFD as this would expose patients to a “poor-quality TTM.” Other relevant aspects on the selection of TTM methods, such the reduced workload of the nursing team and the feasibility of skin counter warming in case of shivering for EC systems when compared to others, should also be assessed in future studies.

Another major issue of this study is the inclusion of different methods (i.e., EC, peritoneal lavage, dialysis techniques) in the same group (i.e., “core”). Also, in some studies, specific TTM methods were combined, in a variable proportion of patients, with cold fluids or iced packs; the role of such additional interventions on the measured outcome remains unknown. However, this allowed the selection of all available studies in the literature comparing at least two different methods of TTM, with a more complete assessment of the study hypothesis. Also, some additional data, such as side effects or speed and precision of cooling, were not collected. However, the time from arrest to target temperature was not further analyzed as this information would be largely biased by the retrospective data collection of most of the studies included in this report. Similarly, the issue of the adverse events has already been discussed in another study [5]. Also, the performance for each TTM method was not routinely reported in all studies. Finally, we could not calculate the minimal number of patients to be included in the meta-analysis, and most of the studies had a high risk of bias, which would reduce the robustness of our findings.

## Conclusions

In this meta-analysis, specific TTM methods (i.e., core, invasive, and with TFD) were associated with a lower probability of poor neurological outcome when compared to other methods in adult CA survivors. However, most of the existing literature is based on retrospective or prospective studies, with a high risk of bias, suggesting new directions for future trials.

## Additional file

Additional file 1: ** Table S1.** Extracted data in each study assessed for eligibility. **Table S2.** Full text articles excluded, not fitting eligibility criteria. **Figure S1.** and **S2.** Funnel plot for studies comparing the impact of core and surface methods on poor neurological outcome (left) and mortality (right). The outer dashed lines indicate the triangular region within which 95% of studies are expected to lie in the absence of biases and heterogeneity. The solid vertical line corresponds to no intervention effect. **Figure S3.** Forest plot of mortality in randomized clinical trials (RCTs) or non-RCTs: invasive vs. non-invasive TTM methods. Size of squares for risk ratio reflects weight of trial in pooled analysis. Horizontal bars represent 95% confidence intervals. **Figure S4.** and **S5.** Funnel plot for studies comparing the impact of invasive and non-invasive TTM methods on poor neurological outcome (left) and mortality (right). The outer dashed lines indicate the triangular region within which 95% of studies are expected to lie in the absence of biases and heterogeneity. The solid vertical line corresponds to no intervention effect. **Figure S6.** Forest plot of mortality in randomized clinical trials (RCTs) or non-RCTs: temperature feedback device (TFD) vs. non-TFD TTM methods. Size of squares for risk ratio reflects weight of trial in pooled analysis. Horizontal bars represent 95% confidence intervals. **Figure S7.** and **S8.** Funnel plot for studies comparing the impact of temperature feedback device (TFD) and non-TFD TTM methods on poor neurological outcome (left) and mortality (right). The outer dashed lines indicate the triangular region within which 95% of studies are expected to lie in the absence of biases and heterogeneity. The solid vertical line corresponds to no intervention effect. **Figure S9.** Forest plot of poor neurological outcome in randomized clinical trials (RCTs) or non-RCTs: endovascular devices vs. air- or water-circulating blankets. Size of squares for risk ratio reflects weight of trial in pooled analysis. Horizontal bars represent 95% confidence intervals.** Figure S10.** Forest plot of mortality in randomized clinical trials (RCTs) or non-RCTs: endovascular devices vs. air- or water-circulating blankets. Size of squares for risk ratio reflects weight of trial in pooled analysis. Horizontal bars represent 95% confidence intervals. **Figure S11.** and **S12.** Funnel plot for studies comparing the impact of endovascular devices vs. air- or water-circulating blankets on poor neurological outcome (left) and mortality (right). The outer dashed lines indicate the triangular region within which 95% of studies are expected to lie in the absence of biases and heterogeneity. The solid vertical line corresponds to no intervention effect. **Figure S13.** Forest plot of poor neurological outcome in randomized clinical trials (RCTs) or non-RCTs: endovascular devices vs. air- or water-circulating blankets with temperature feedback device (TFD). Size of squares for risk ratio reflects weight of trial in pooled analysis. Horizontal bars represent 95% confidence intervals. **Figure S14.** Forest plot of mortality in randomized clinical trials (RCTs) or non-RCTs: endovascular devices vs. air- or water-circulating blankets with temperature feedback device (TFD). Size of squares for risk ratio reflects weight of trial in pooled analysis. Horizontal bars represent 95% confidence intervals. **Figure S15.** and **S16.** Funnel plot for studies comparing the impact of endovascular devices vs. air- or water-circulating blankets with temperature feedback device (TFD) on poor neurological outcome (left) and mortality (right). The outer dashed lines indicate the triangular region within which 95% of studies are expected to lie in the absence of biases and heterogeneity. The solid vertical line corresponds to no intervention effect. **Figure S17.** Forest plot of poor neurological outcome in randomized clinical trials (RCTs) or non-RCTs: blankets vs. other surface TTM methods. Size of squares for risk ratio reflects weight of trial in pooled analysis. Horizontal bars represent 95% confidence intervals. **Figure S18.** Forest plot of mortality in randomized clinical trials (RCTs) or non-RCTs: blankets vs. other surface TTM methods. Size of squares for risk ratio reflects weight of trial in pooled analysis. Horizontal bars represent 95% confidence intervals. **Figure S19.** and **S20.** Funnel plot for studies comparing the impact of blankets vs. other surface TTM methods on poor neurological outcome (left) and mortality (right). The outer dashed lines indicate the triangular region within which 95% of studies are expected to lie in the absence of biases and heterogeneity. The solid vertical line corresponds to no intervention effect. (DOCX 6046 kb)

## Data Availability

The datasets used and/or analyzed during the current study are available from the corresponding author on reasonable request.

## Abbreviations

CA: Cardiac arrest
CPC: Cerebral performance category
GCS: Glasgow Coma Score
EC: Endovascular catheter
OHCA: Out-of-hospital cardiac arrest
RCT: Randomized clinical trial
ROSC: Return of spontaneous circulation
TFD: Temperature feedback device
TTM: Targeted temperature management
