# Supplementary material for: Effect of different methods of cooling for targeted temperature management on outcome after cardiac arrest: a systematic review and meta-analysis
Source: Crit Care. 2019 Aug 23;23:285. doi: 10.1186/s13054-019-2567-6 (PMC6708171; doi:10.1186/s13054-019-2567-6)

**SUPPLEMENTARY MATERIAL**

**Characteristics of the selected studies (Table S1 in the main manuscript).**

Thirteen studies (59%) included only patients after OHCA; target temperature during TTM was 32-34°C in all studies except one, which had patients treated either at 33°C or 36°C [14]. Duration of cooling was 24 hours in 20 studies (91%); one study reported TTM duration of 12-24 hours [32] and another compared TTM during 24 hours vs 48 hours [17]. Poor neurological outcome was reported in 14 studies (64%) and defined as a Cerebral Performance Category of 3-5 in all of them; in 7 of these studies (50%), neurological outcome was collected at hospital discharge. Mortality was reported in all studies; most studies reported mortality at hospital discharge (12/22, 55%) and only 3 studies reported long-term mortality at 6 months after CA.

**Table S1.** Extracted data in each study assessed for eligibility

| **Extracted Data Details** |
| --- |

Study Reference Names and surnames of authors, year of publication

Study design Type of recruitment

Patient number Number of eligible patients in each study

Cardiac Arrest Location OHCA or both IHCA and OHCA

Target Temperature during Hypothermia i.e. 32-34°C or others

Duration of cooling i.e. 12, 24 or 48 hours

Timing of reported mortality i.e. ICU discharge, Hospital discharge or others

Timing of reported poor neurological outcome i.e. ICU discharge, Hospital discharge or others

Type of TTM methods i.e. endovascular, blankets, ice packs or others

Definition of TTM methods i.e. core vs. surface (see *Methods*)

Non-survivors Number of dead patients in each group

Poor neurological outcome Number of patients with poor neurological outcome in each group

**Main Research Questions**: a) Can neurological outcome of patients resuscitated after cardiac arrest be influenced by the method used for targeted temperature management (TTM) ?

b) Can survival of patients resuscitated after cardiac arrest be influenced by the method used for targeted temperature management (TTM) ?

**PICOS**

Participants Patients resuscitated from cardiac arrest undergoing targeted temperature management (TTM)

Interventions Targeted temperature management (TTM)

Comparisons At least two different TTM methods

Outcomes Neurological Outcome; Mortality

Study design Retrospective, Prospective and Randomized

______________________________________________________________________________

OHCA = out-of-hospital cardiac arrest; IHCA = in-hospital cardiac arrest; ICU = intensive care unit

**Table S2.** Full text articles excluded, not fitting eligibility criteria.

| **Excluded Studies Main reason for exclusion** |
| --- |

Holzer et al. 2006 Normothermic control group.

Fink et al. 2008 Article in German

Lin et al. 2014 Normothermic control group.

Hegazy et al. 2017 Case report.

Markota et al. 2016 No control group.

Grave et al. 2016 No control group.

Bernard et al. 2016 Normothermic control group.

Polderman et al. 2015 No control group.

Kliegel et al. 2005 No control group.

Karacan et al. 2009 Article in Danish.

Kamarainen et al. 2009 Normothermic control group.

De Bourmont et al. 2015 No control group.

Knapik et al. 2011 Full article not found.

Castren et al. 2010 Intra-arrest cooling.

Takeda et al. 2014 Mixed cooling.

Uray et al. 2010 Mixed cooling.

De Waard et al. 2013 Duplicate.

Shinada et al. 2014 Duplicate.

Rana et al. 2011 Duplicate.

Heard et al. 2010 Duplicate.

Laurent et al. 2005 Normothermic control group.

Nielsen et al. 2009 Mixed cooling.

Islam et al. 2015 Partial cooling (induction).

Hoedemakers et al. 2007 Mixed population (not just cardiac arrest patients).

___________________________________________________________________________________________

**Figure S1 and S2:** Funnel plot for studies comparing the impact of core and surface methods on poor neurological outcome (left) and mortality (right). The outer dashed lines indicate the triangular region within which 95% of studies are expected to lie in the absence of biases and heterogeneity. The solid vertical line corresponds to no intervention effect.


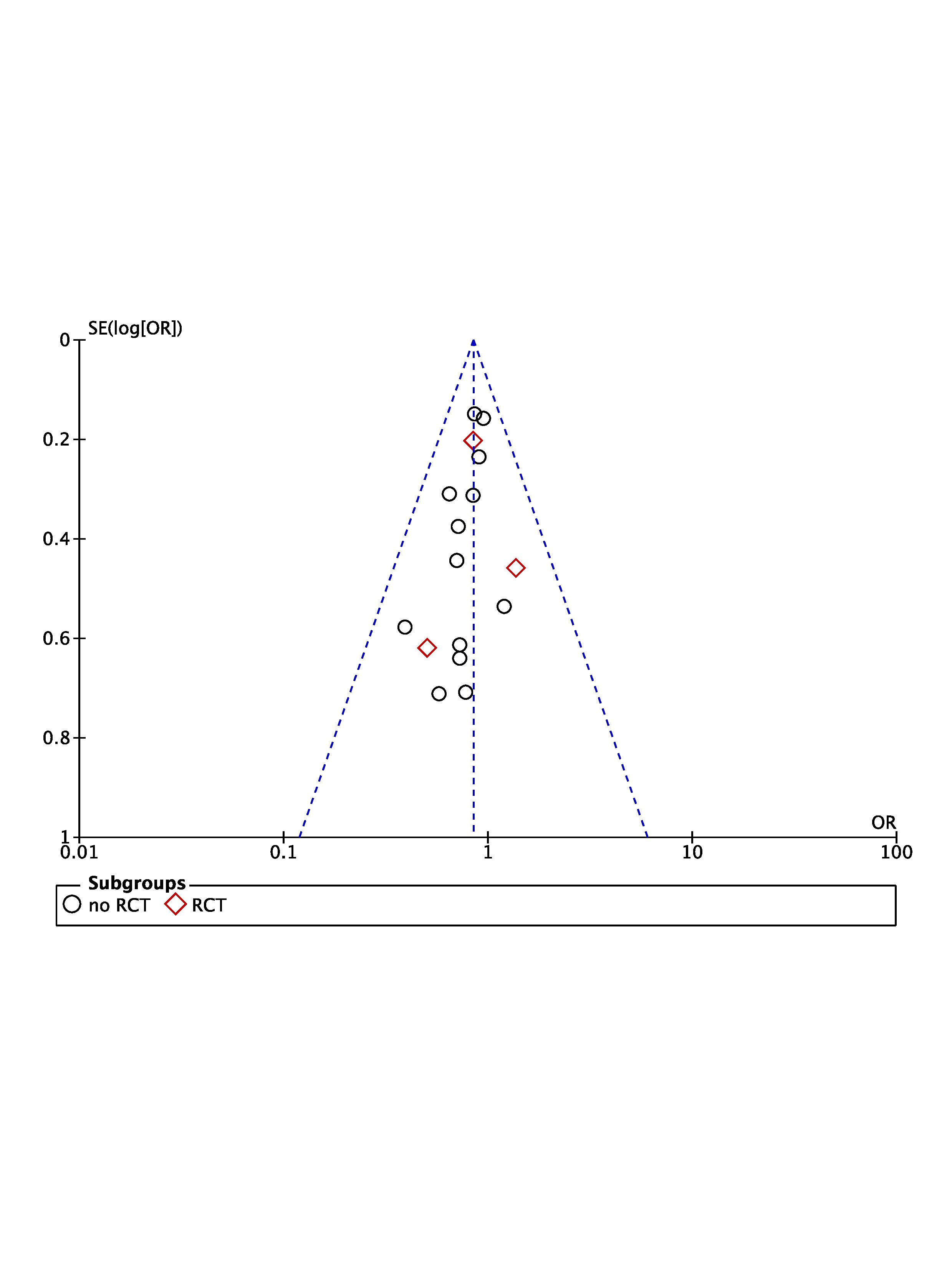


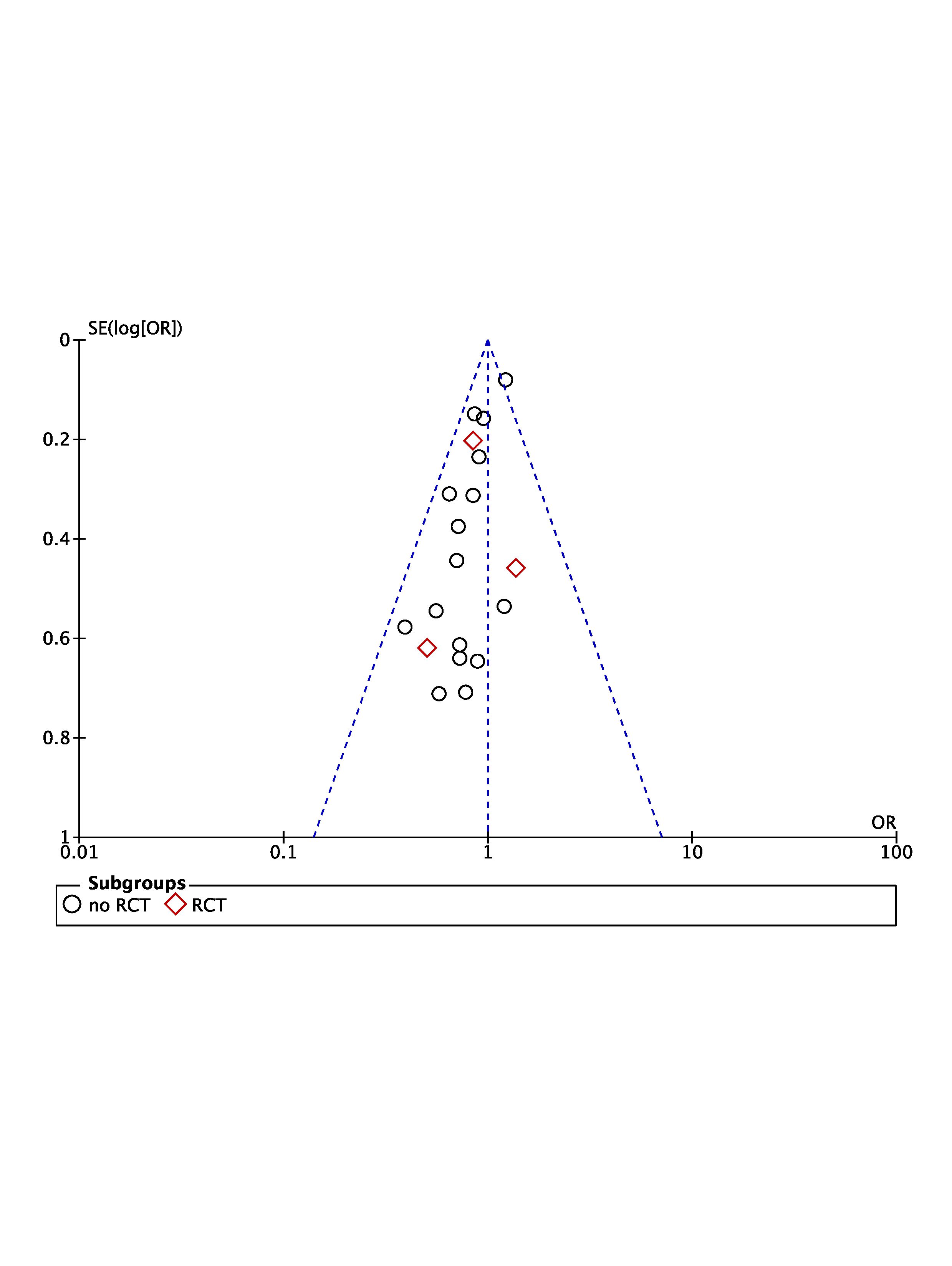


**Figure S3:** Forest plot of mortality in randomized clinical trials (RCTs) or non-RCTs: invasive vs. non-invasive TTM methods. Size of squares for risk ratio reflects weight of trial in pooled analysis. Horizontal bars represent 95% confidence intervals.


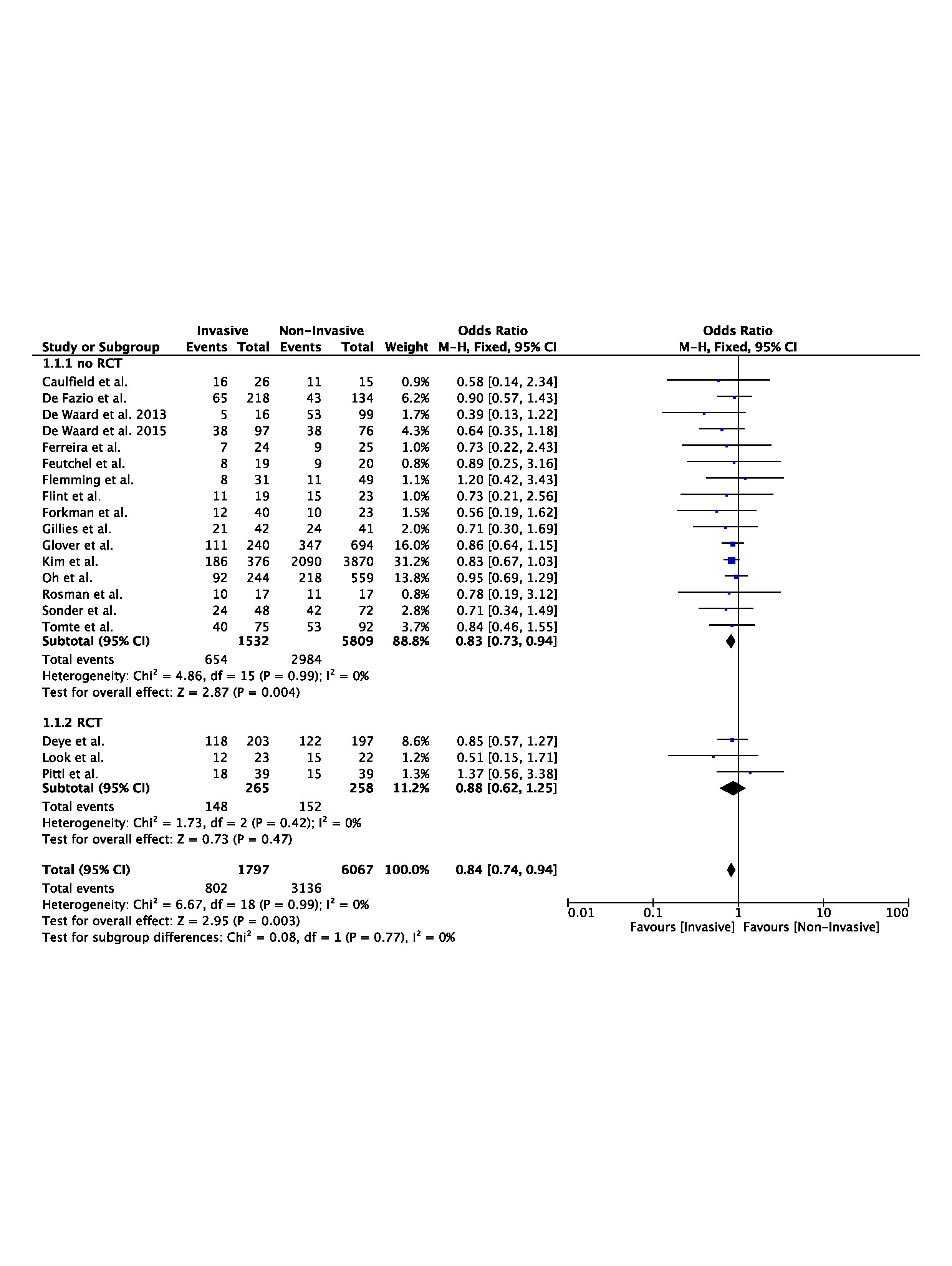


**Figure S4 and S5:** Funnel plot for studies comparing the impact of invasive and non-invasive TTM methods on poor neurological outcome (left) and mortality (right). The outer dashed lines indicate the triangular region within which 95% of studies are expected to lie in the absence of biases and heterogeneity. The solid vertical line corresponds to no intervention effect.


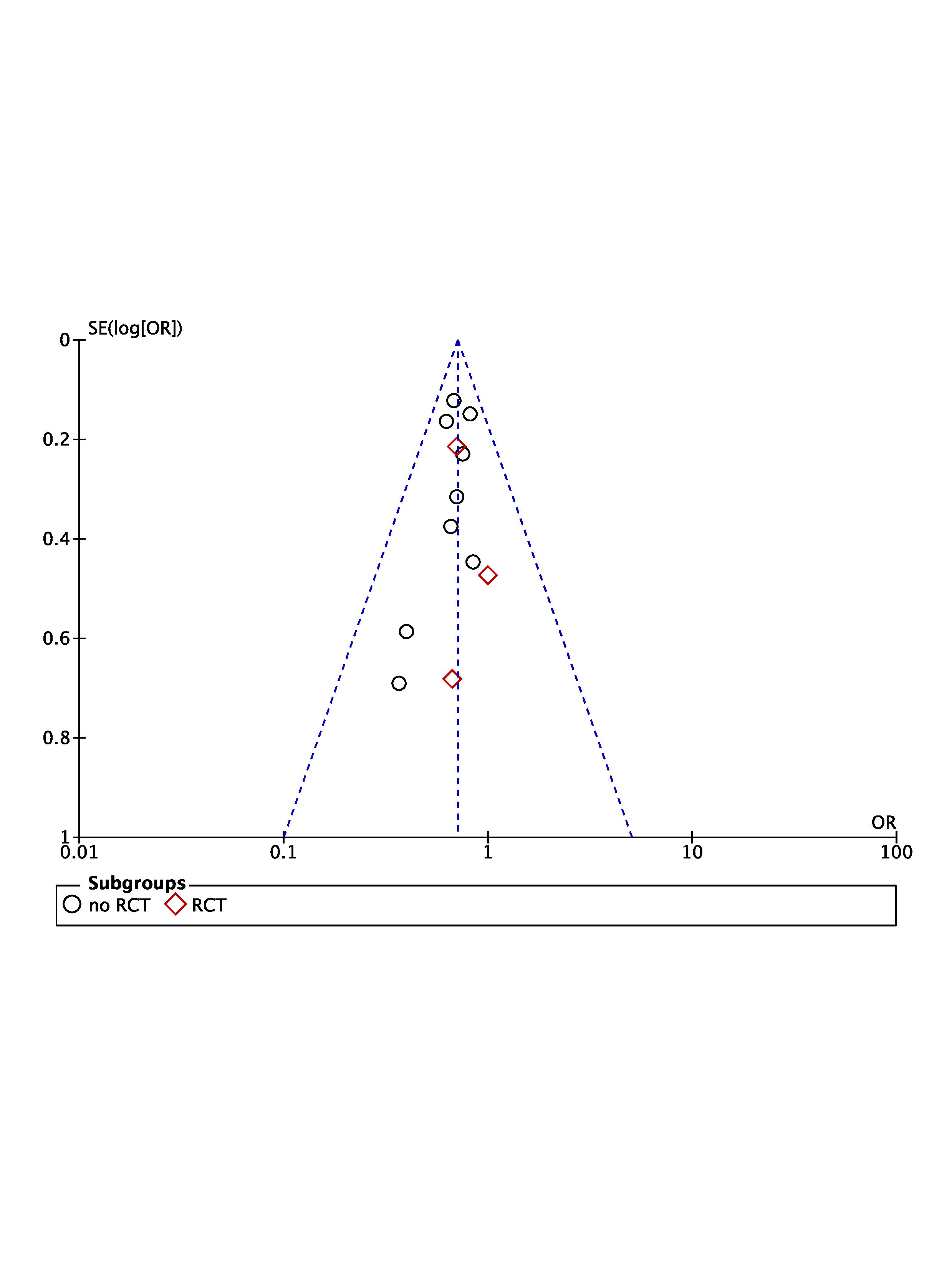

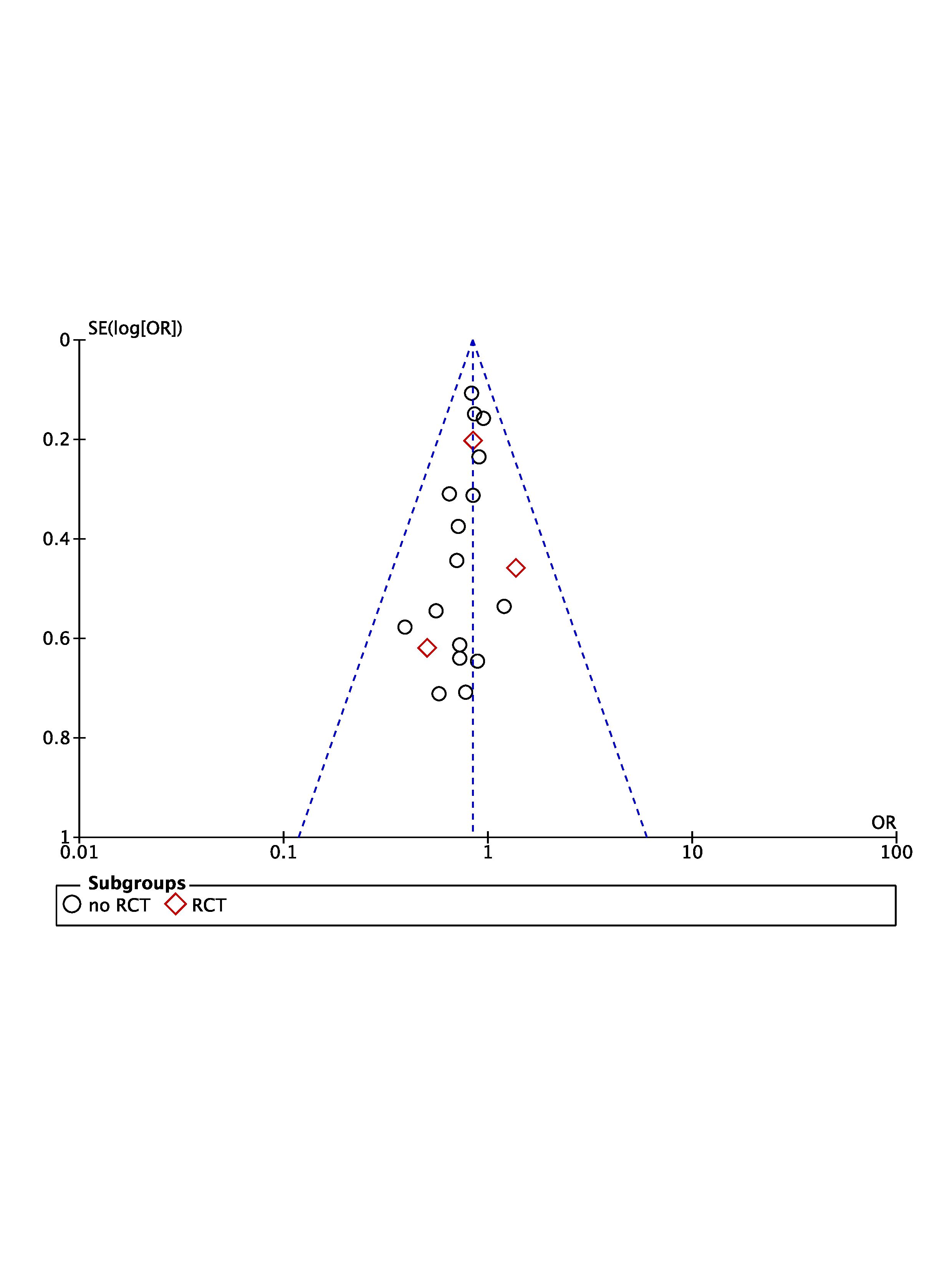


**Figure S6:** Forest plot of mortality in randomized clinical trials (RCTs) or non-RCTs: temperature feedback device (TFD) vs. non-TFD TTM methods. Size of squares for risk ratio reflects weight of trial in pooled analysis. Horizontal bars represent 95% confidence intervals.


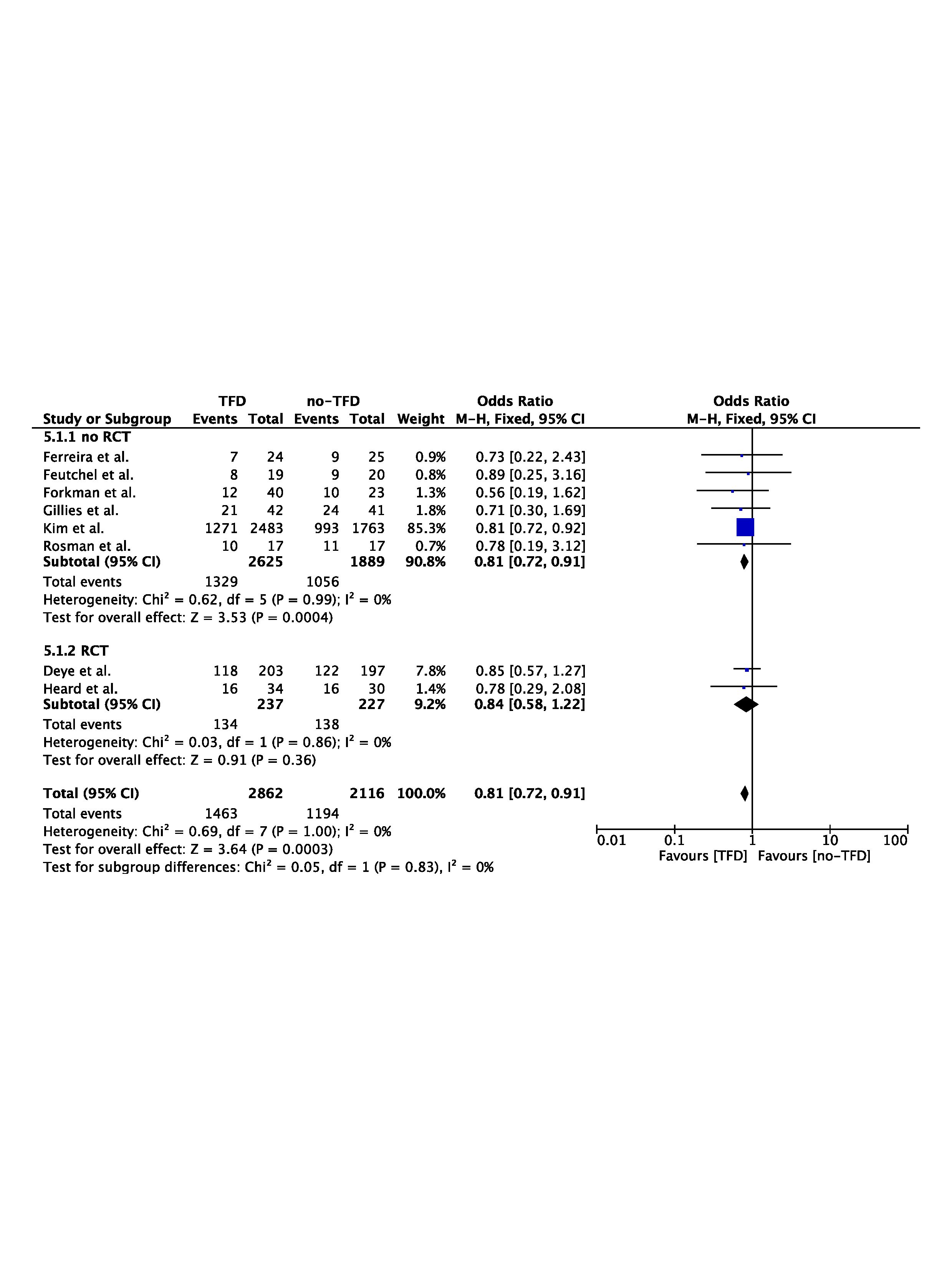


**Figure S7 and S8:** Funnel plot for studies comparing the impact of temperature feedback device (TFD) and non-TFD TTM methods on poor neurological outcome (left) and mortality (right). The outer dashed lines indicate the triangular region within which 95% of studies are expected to lie in the absence of biases and heterogeneity. The solid vertical line corresponds to no intervention effect.


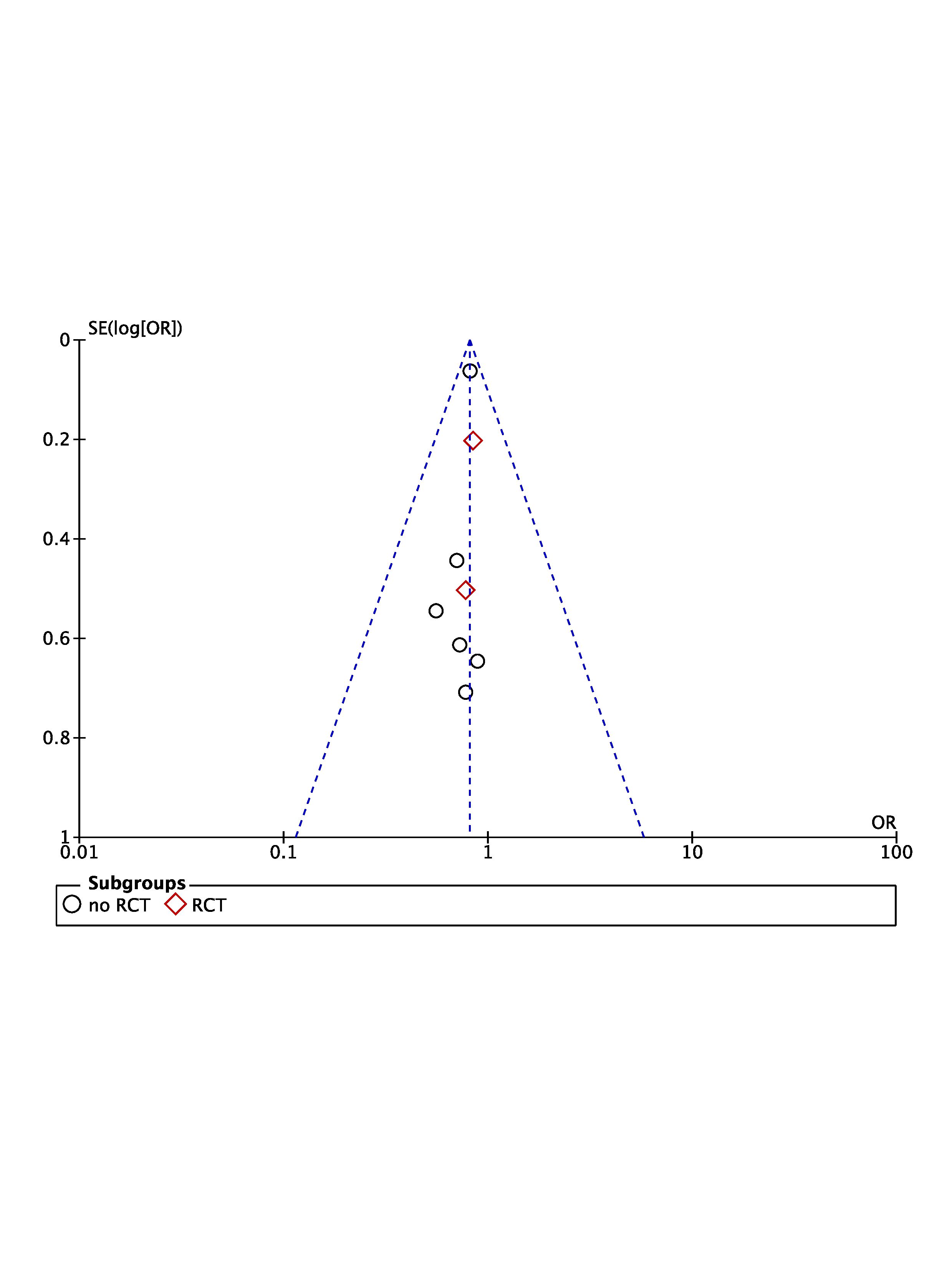


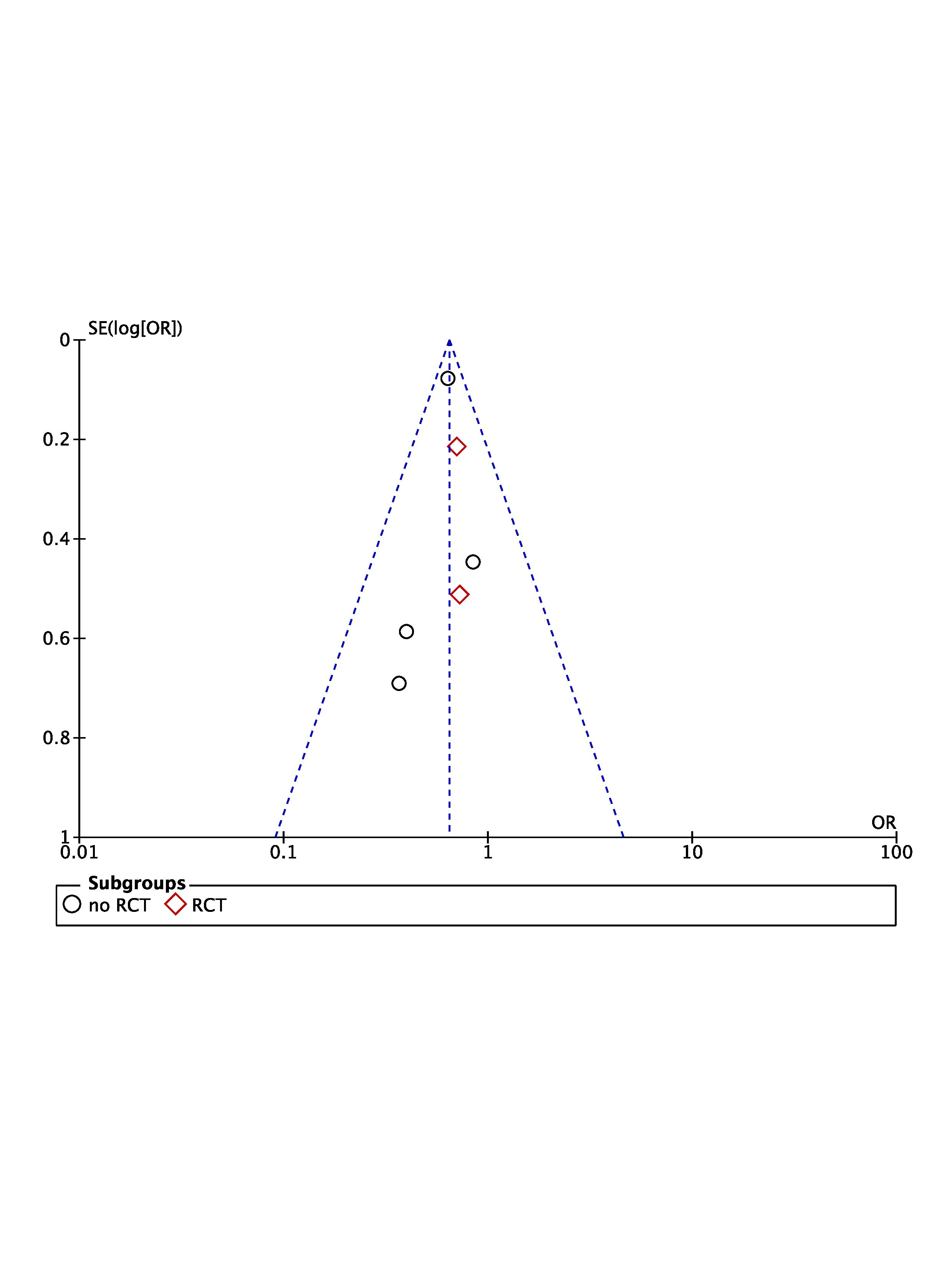


**Figure S9:** Forest plot of poor neurological outcome in randomized clinical trials (RCTs) or non-RCTs: endovascular devices vs. air- or water-circulating blankets. Size of squares for risk ratio reflects weight of trial in pooled analysis. Horizontal bars represent 95% confidence intervals.


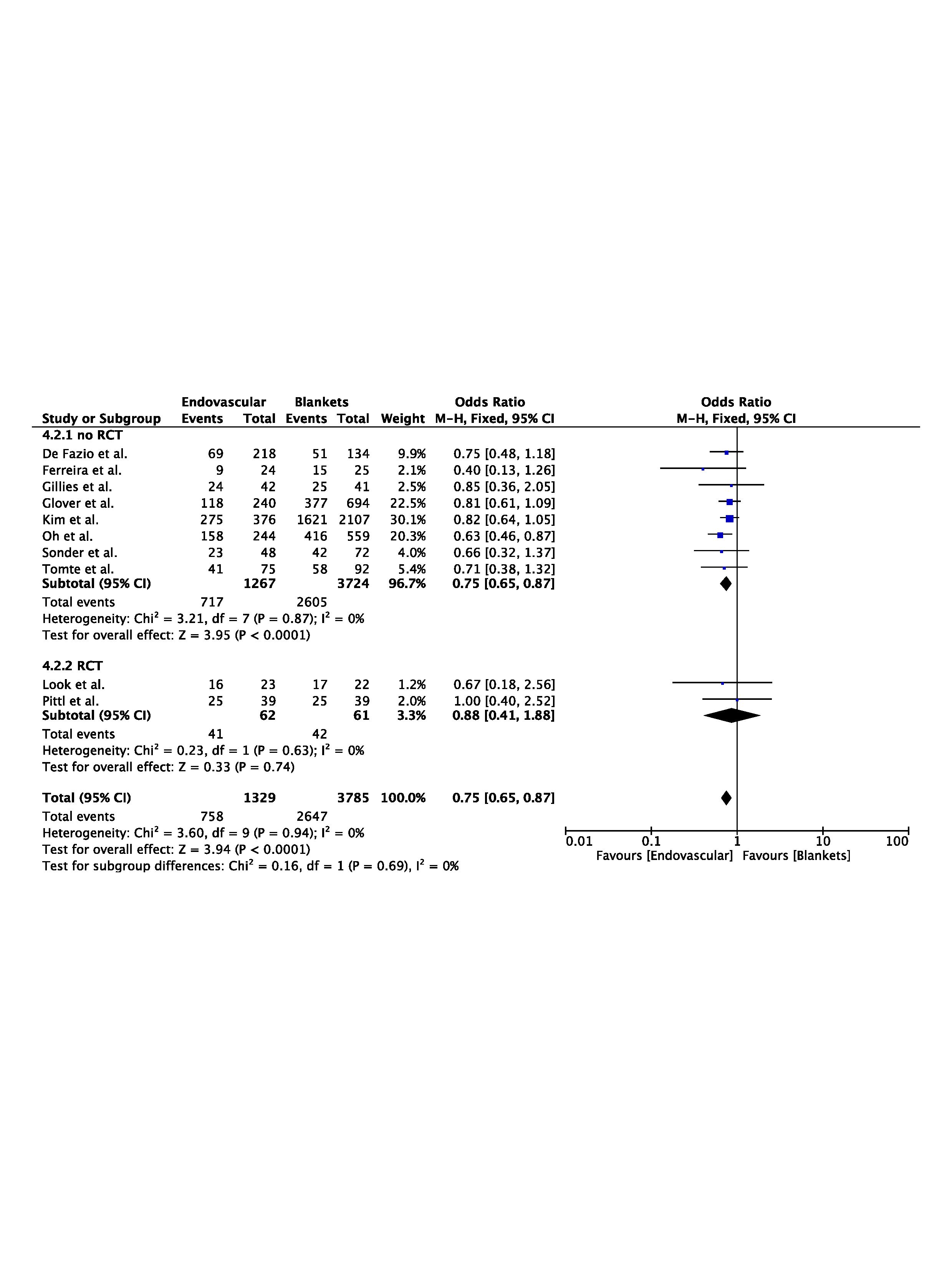


**Figure S10:** Forest plot of mortality in randomized clinical trials (RCTs) or non-RCTs: endovascular devices vs. air- or water-circulating blankets. Size of squares for risk ratio reflects weight of trial in pooled analysis. Horizontal bars represent 95% confidence intervals.


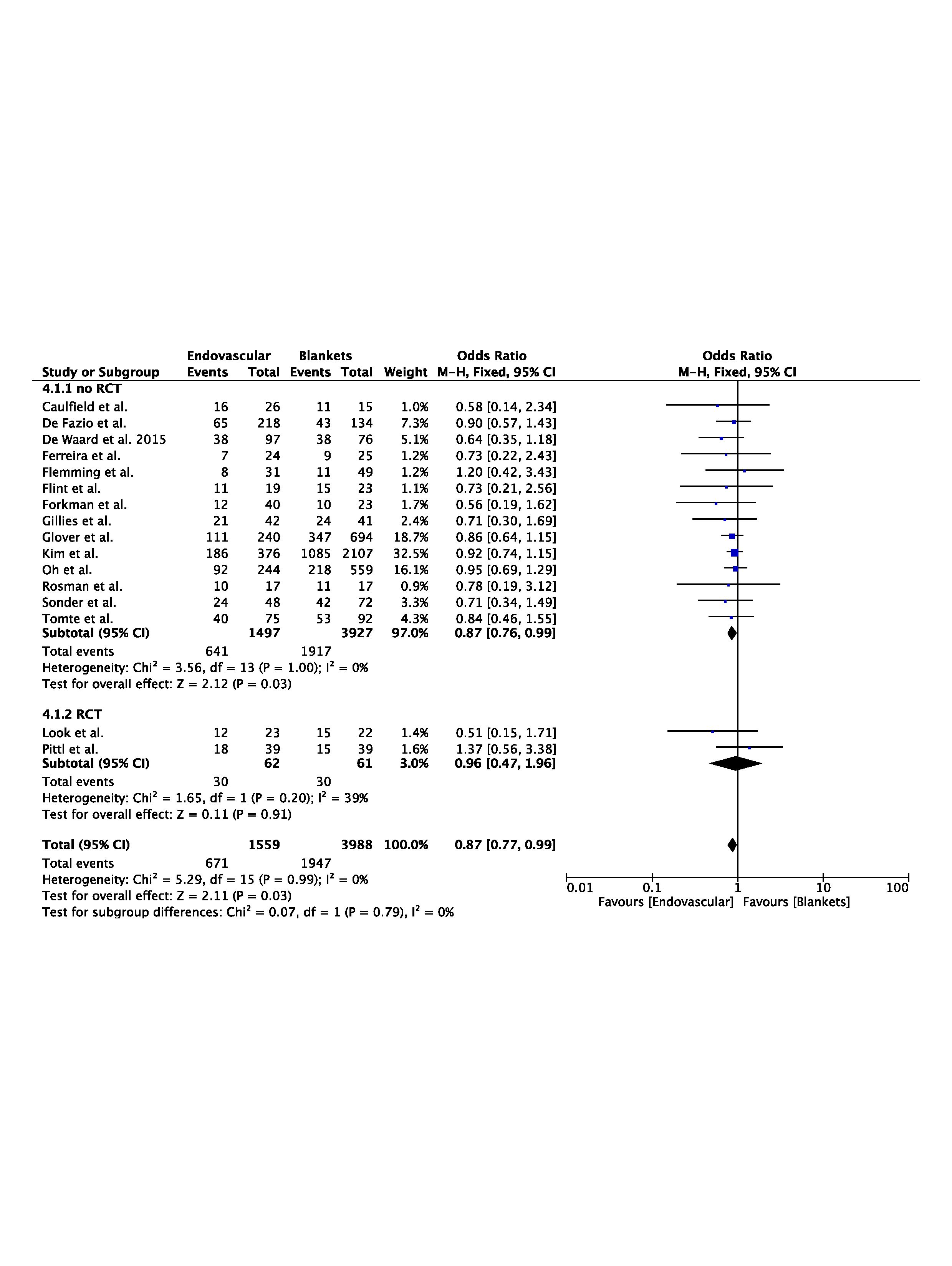


**Figure S11 and S12:** Funnel plot for studies comparing the impact of endovascular devices vs. air- or water-circulating blankets on poor neurological outcome (left) and mortality (right). The outer dashed lines indicate the triangular region within which 95% of studies are expected to lie in the absence of biases and heterogeneity. The solid vertical line corresponds to no intervention effect.


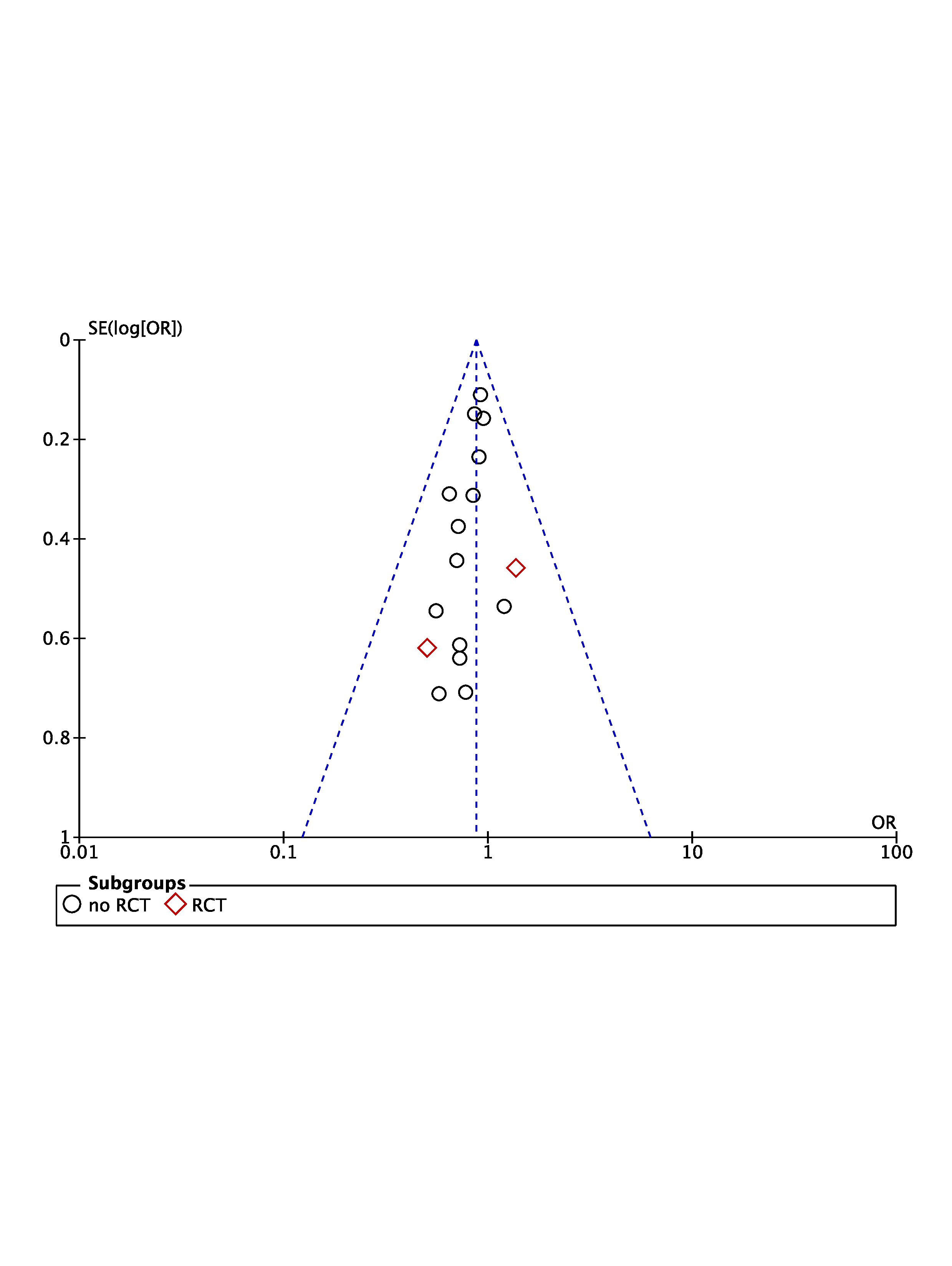

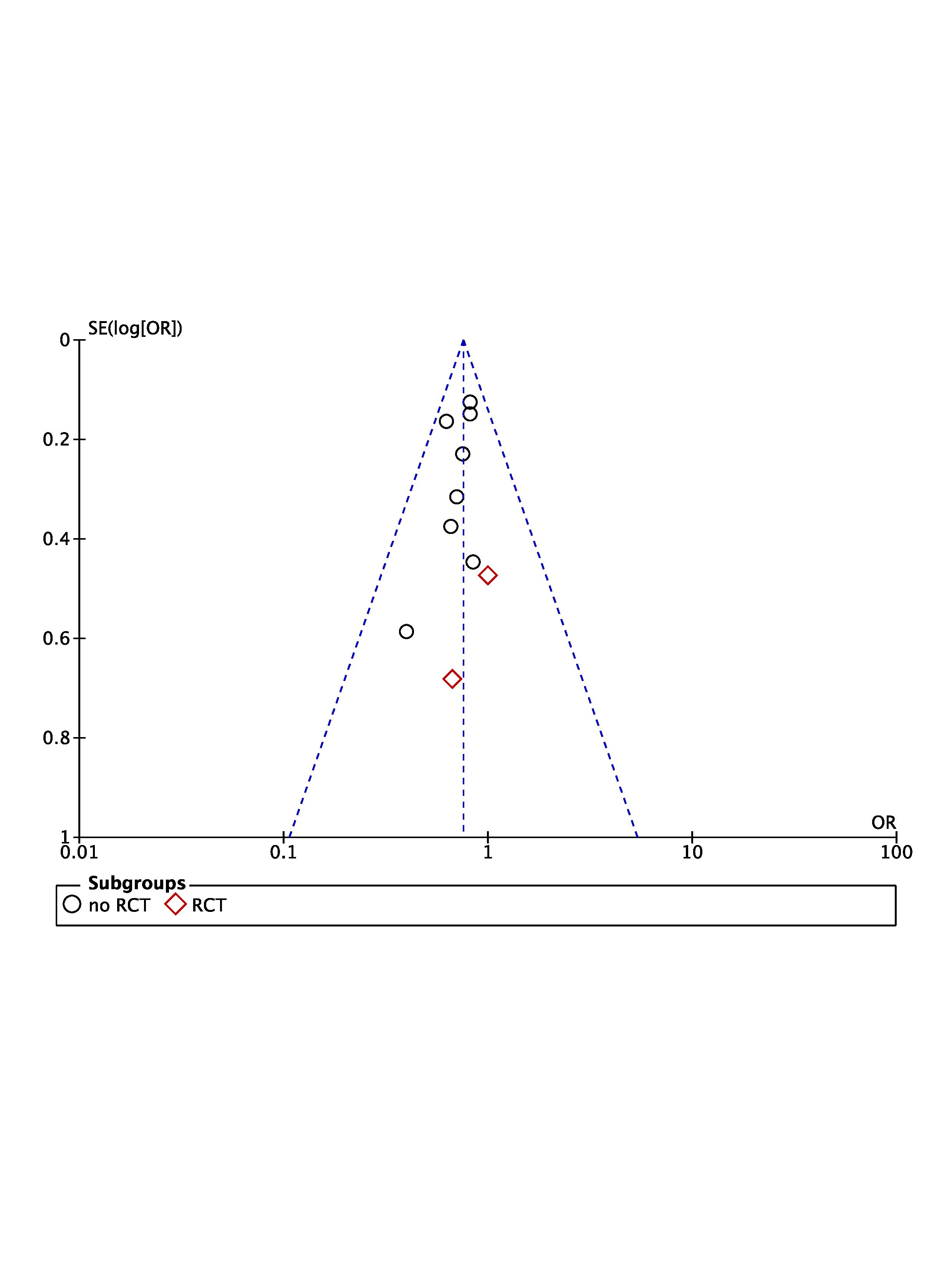


**Figure S13:** Forest plot of poor neurological outcome in randomized clinical trials (RCTs) or non-RCTs: endovascular devices vs. air- or water-circulating blankets with temperature feedback device (TFD). Size of squares for risk ratio reflects weight of trial in pooled analysis. Horizontal bars represent 95% confidence intervals.


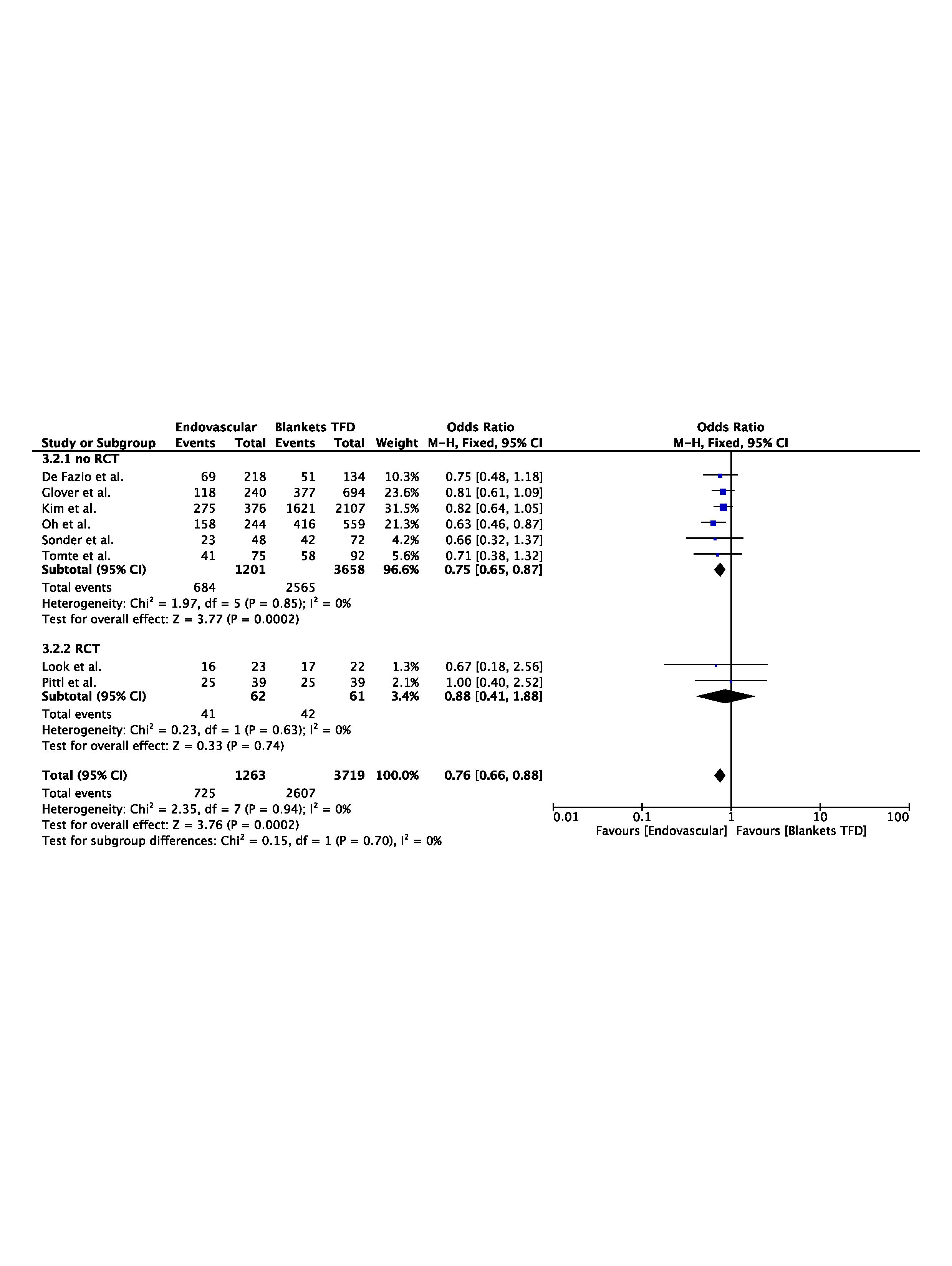


**Figure S14:** Forest plot of mortality in randomized clinical trials (RCTs) or non-RCTs: endovascular devices vs. air- or water-circulating blankets with temperature feedback device (TFD). Size of squares for risk ratio reflects weight of trial in pooled analysis. Horizontal bars represent 95% confidence intervals.


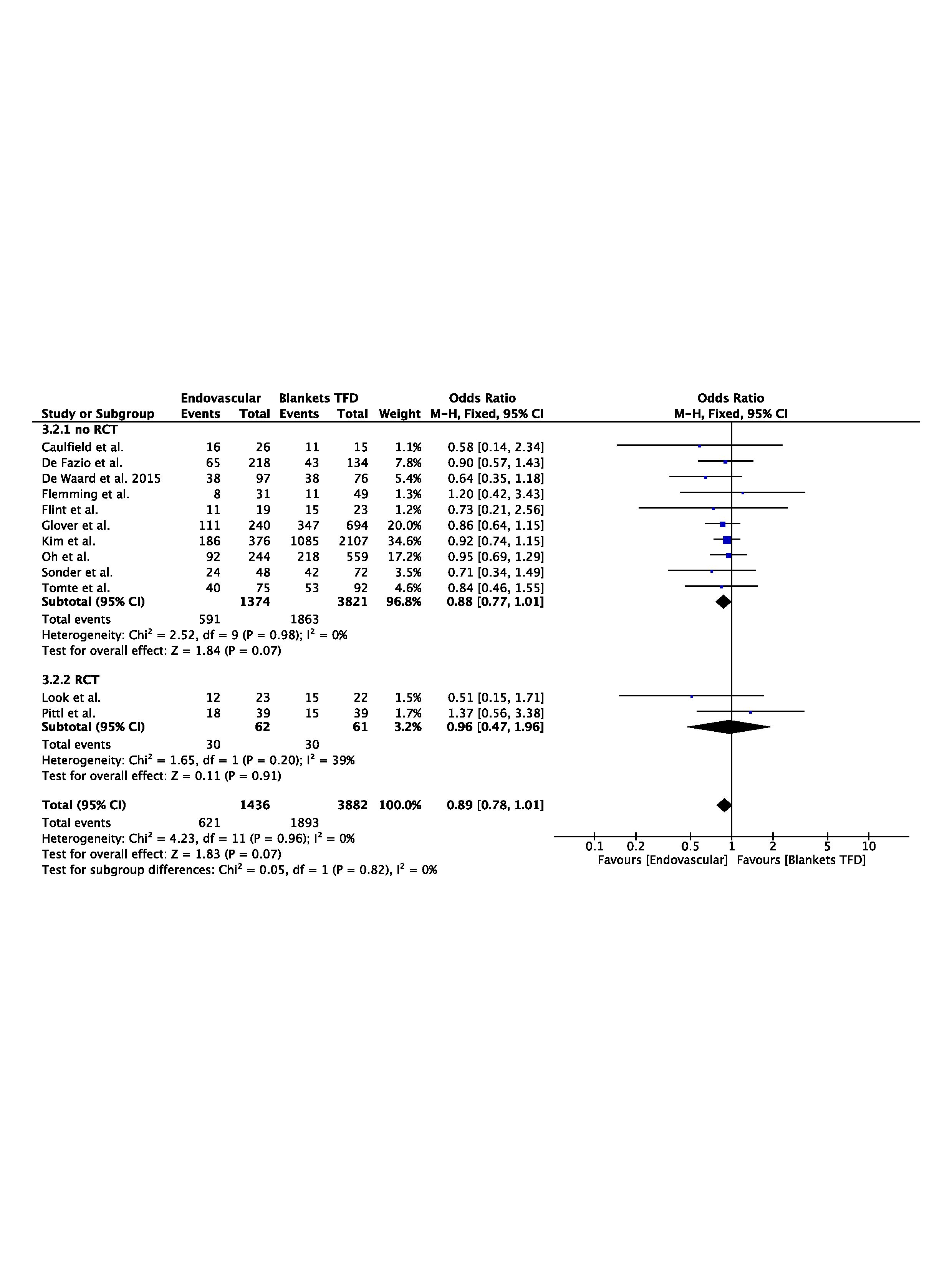


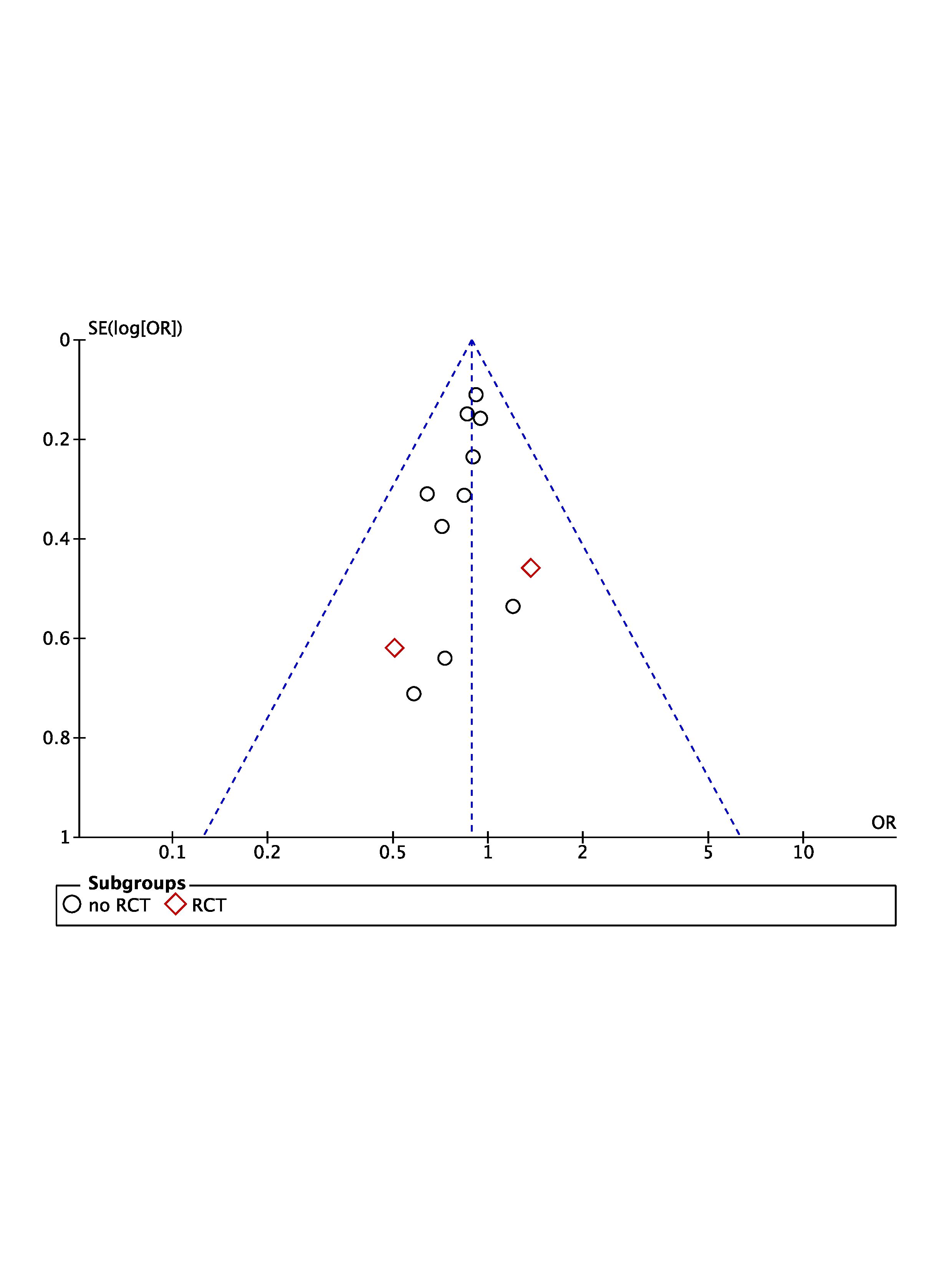
**Figure S15 and S16:** Funnel plot for studies comparing the impact of endovascular devices vs. air- or water-circulating blankets with temperature feedback device (TFD) on poor neurological outcome (left) and mortality (right). The outer dashed lines indicate the triangular region within which 95% of studies are expected to lie in the absence of biases and heterogeneity. The solid vertical line corresponds to no intervention effect.


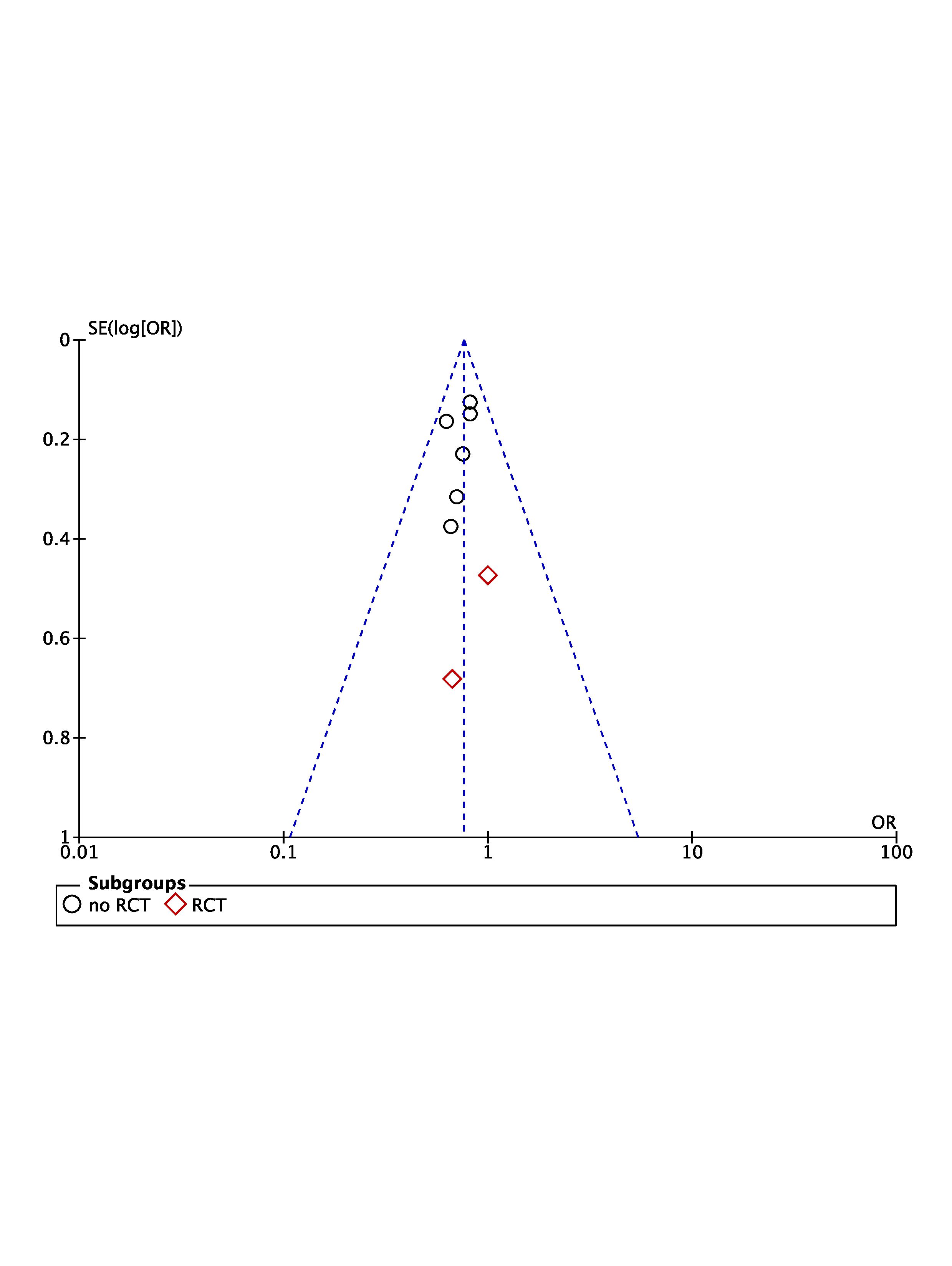


**Figure S17:** Forest plot of poor neurological outcome in randomized clinical trials (RCTs) or non-RCTs: blankets vs. other surface TTM methods. Size of squares for risk ratio reflects weight of trial in pooled analysis. Horizontal bars represent 95% confidence intervals.


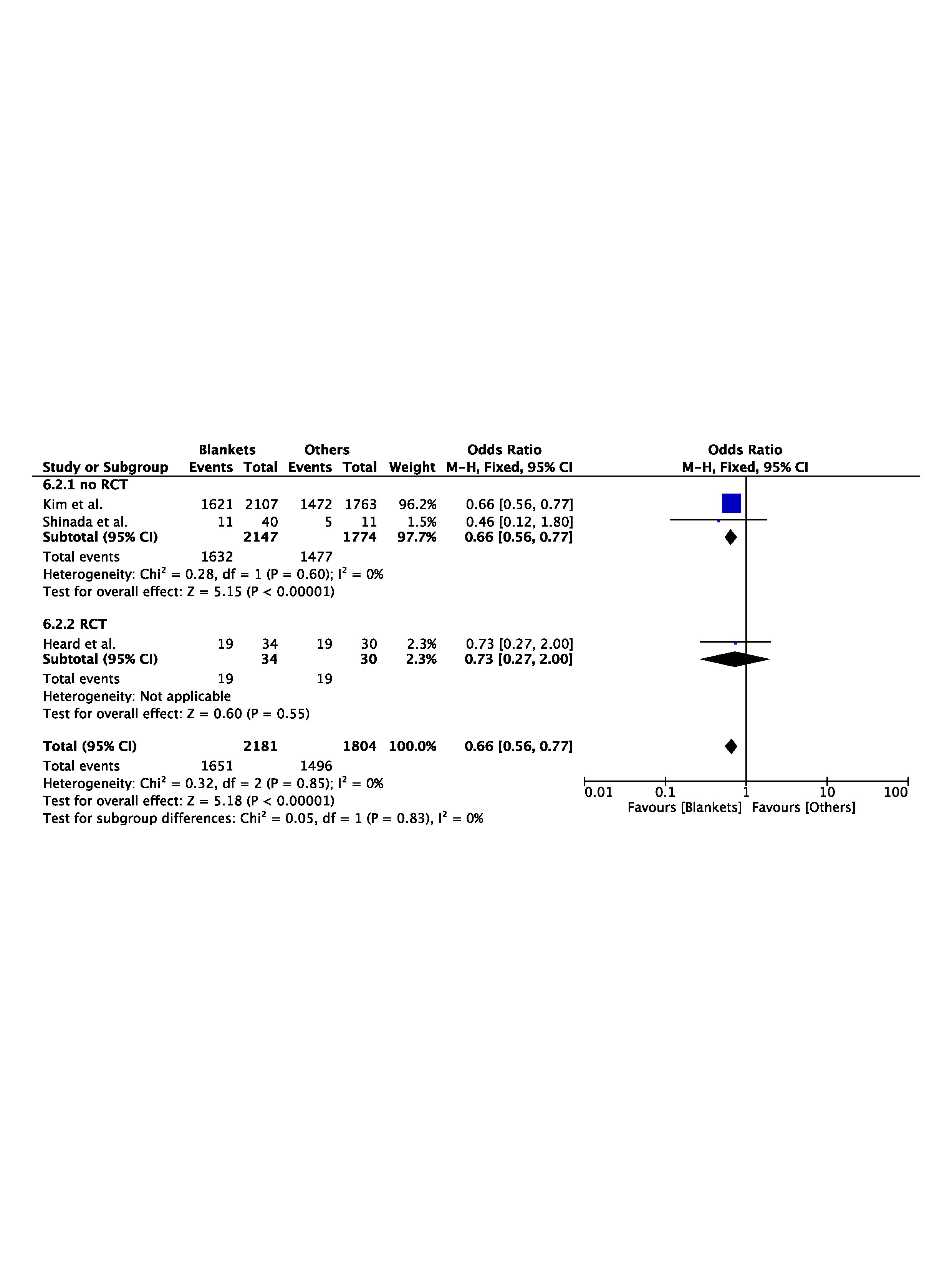


**Figure S18:** Forest plot of mortality in randomized clinical trials (RCTs) or non-RCTs: blankets vs. other surface TTM methods. Size of squares for risk ratio reflects weight of trial in pooled analysis. Horizontal bars represent 95% confidence intervals.


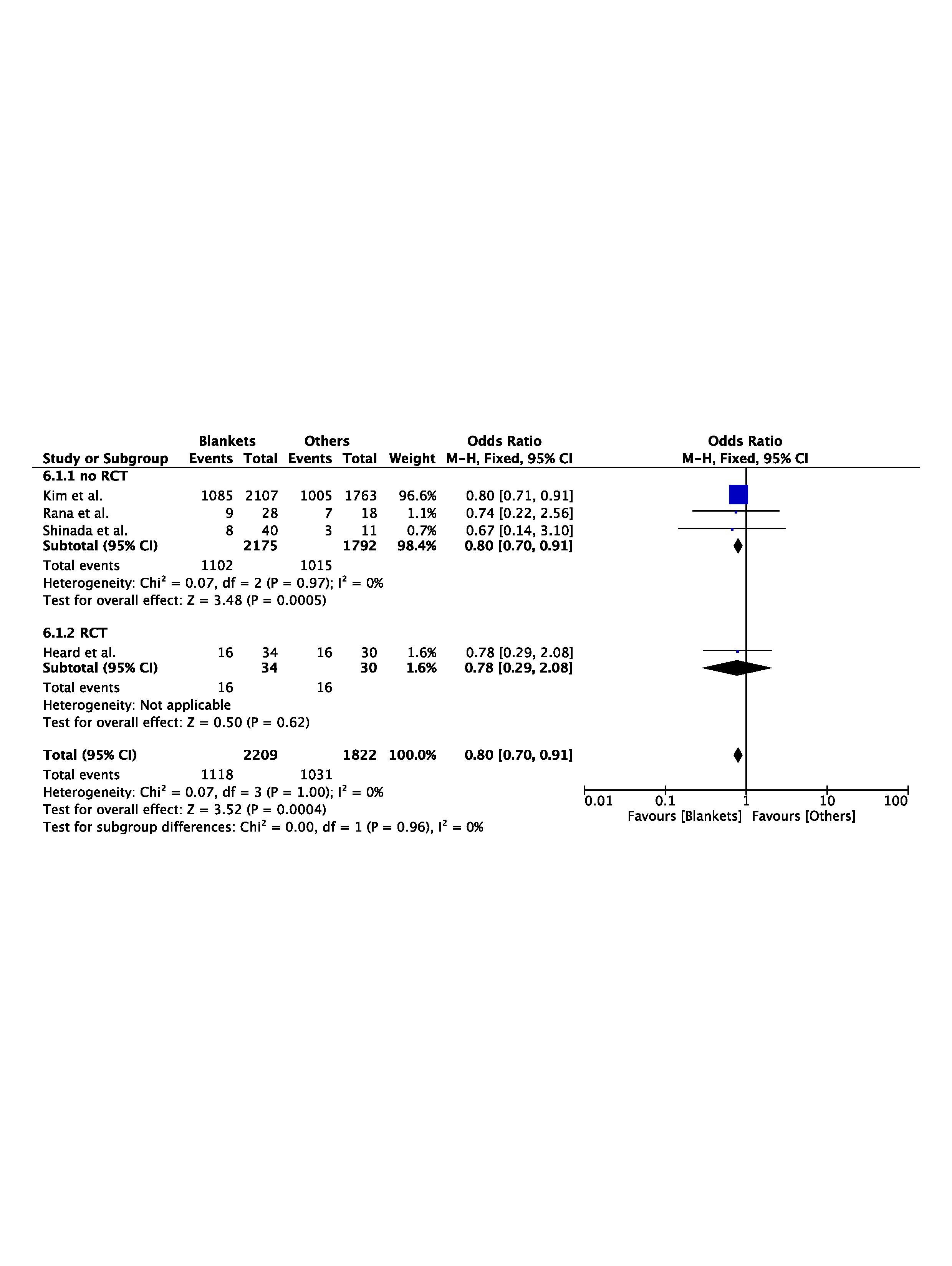


**Figure S19 and S20:** Funnel plot for studies comparing the impact of blankets vs. other surface TTM methods on poor neurological outcome (left) and mortality (right). The outer dashed lines indicate the triangular region within which 95% of studies are expected to lie in the absence of biases and heterogeneity. The solid vertical line corresponds to no intervention effect.


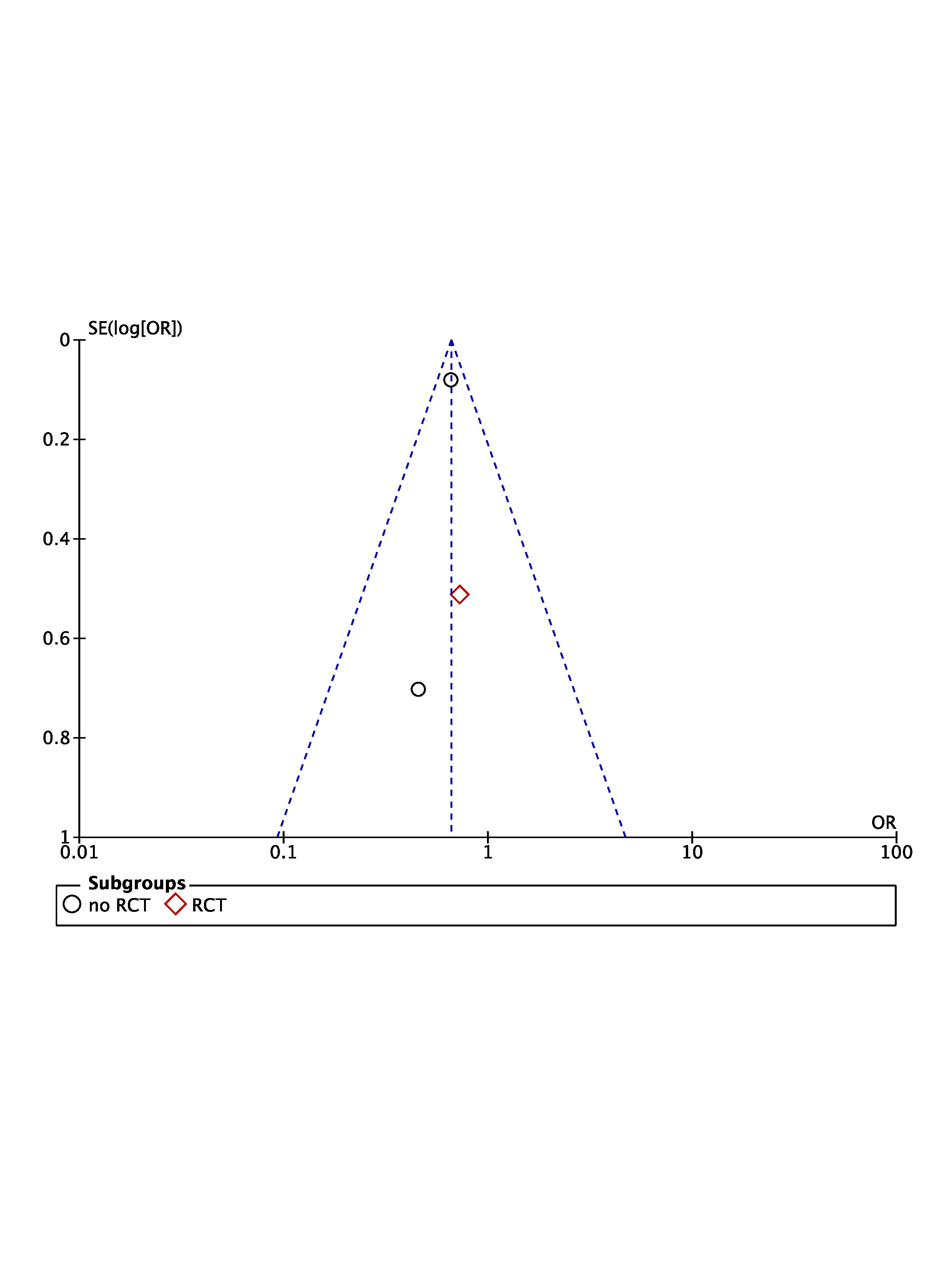

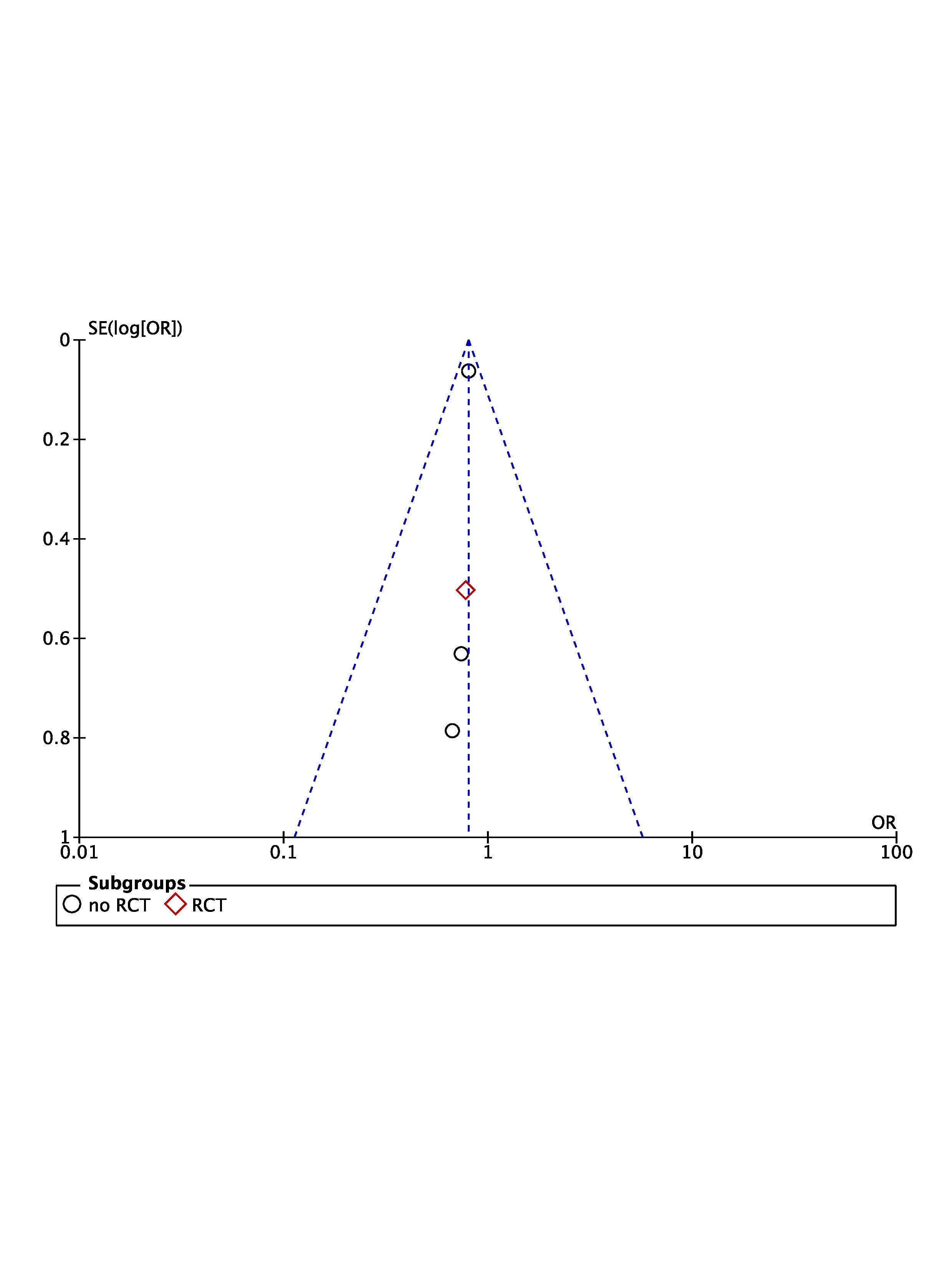

Supplement: Supplementary file 1 — Table S1. Extracted data in each study assessed for eligibility. Table S2. Full text articles excluded, not fitting eligibility criteria. Figure S1. and S2. Funnel plot for studies comparing the impact of core and surface methods on poor neurological outcome (left) and mortality (right). The outer dashed lines indicate the triangular region within which 95% of studies are expected to lie in the absence of biases and heterogeneity. The solid vertical line corresponds to no intervention effect. Figure S3. Forest plot of mortality in randomized clinical trials (RCTs) or non-RCTs: invasive vs. non-invasive TTM methods. Size of squares for risk ratio reflects weight of trial in pooled analysis. Horizontal bars represent 95% confidence intervals. Figure S4. and S5. Funnel plot for studies comparing the impact of invasive and non-invasive TTM methods on poor neurological outcome (left) and mortality (right). The outer dashed lines indicate the triangular region within which 95% of studies are expected to lie in the absence of biases and heterogeneity. The solid vertical line corresponds to no intervention effect. Figure S6. Forest plot of mortality in randomized clinical trials (RCTs) or non-RCTs: temperature feedback device (TFD) vs. non-TFD TTM methods. Size of squares for risk ratio reflects weight of trial in pooled analysis. Horizontal bars represent 95% confidence intervals. Figure S7. and S8. Funnel plot for studies comparing the impact of temperature feedback device (TFD) and non-TFD TTM methods on poor neurological outcome (left) and mortality (right). The outer dashed lines indicate the triangular region within which 95% of studies are expected to lie in the absence of biases and heterogeneity. The solid vertical line corresponds to no intervention effect. Figure S9. Forest plot of poor neurological outcome in randomized clinical trials (RCTs) or non-RCTs: endovascular devices vs. air- or water-circulating blankets. Size of squares for risk ratio reflects weight [file 13054_2019_2567_MOESM1_ESM.docx]
